# Targeted *Tshz3* deletion in corticostriatal circuit components segregates core autistic behaviors

**DOI:** 10.1038/s41398-022-01865-6

**Published:** 2022-03-15

**Authors:** Xavier Caubit, Paolo Gubellini, Pierre L. Roubertoux, Michèle Carlier, Jordan Molitor, Dorian Chabbert, Mehdi Metwaly, Pascal Salin, Ahmed Fatmi, Yasmine Belaidouni, Lucie Brosse, Lydia Kerkerian-Le Goff, Laurent Fasano

**Affiliations:** 1 Aix-Marseille Univ, CNRS, IBDM, UMR7288 Marseille, France; 2 Aix-Marseille Univ, INSERM, MMG, UMR1251 Marseille, France; 3 Aix-Marseille Univ, CNRS, LPC, UMR7290 Marseille, France

**Keywords:** Molecular neuroscience, Autism spectrum disorders

## Abstract

We previously linked *TSHZ3* haploinsufficiency to autism spectrum disorder (ASD) and showed that embryonic or postnatal *Tshz3* deletion in mice results in behavioral traits relevant to the two core domains of ASD, namely social interaction deficits and repetitive behaviors. Here, we provide evidence that cortical projection neurons (CPNs) and striatal cholinergic interneurons (SCINs) are two main and complementary players in the TSHZ3-linked ASD syndrome. In the cerebral cortex, TSHZ3 is expressed in CPNs and in a proportion of GABAergic interneurons, but not in cholinergic interneurons or glial cells. In the striatum, TSHZ3 is expressed in all SCINs, while its expression is absent or partial in the other main brain cholinergic systems. We then characterized two new conditional knockout (cKO) models generated by crossing *Tshz3*^*flox/flox*^ with *Emx1-Cre* (*Emx1-cKO*) or *Chat-Cre* (*Chat-cKO*) mice to decipher the respective role of CPNs and SCINs. *Emx1-cKO* mice show altered excitatory synaptic transmission onto CPNs and impaired plasticity at corticostriatal synapses, with neither cortical neuron loss nor abnormal layer distribution. These animals present social interaction deficits but no repetitive patterns of behavior. *Chat-cKO* mice exhibit no loss of SCINs but changes in the electrophysiological properties of these interneurons, associated with repetitive patterns of behavior without social interaction deficits. Therefore, dysfunction in either CPNs or SCINs segregates with a distinct ASD behavioral trait. These findings provide novel insights onto the implication of the corticostriatal circuitry in ASD by revealing an unexpected neuronal dichotomy in the biological background of the two core behavioral domains of this disorder.

## Introduction

Autism spectrum disorder (ASD) includes a heterogeneous group of neurodevelopmental pathologies the diagnosis of which is based exclusively on behavioral criteria. The two behavioral domains that are selected by the DSM-5 are: (i) deficit in social communication and (ii) restrictive, repetitive patterns of behavior, interests, or activities [1]. These domains also emerge from factor analyses of the 13 available diagnostic instruments in patients [2] and in a model that aligns mouse and patient features [3]. More than 900 genes have been liked to ASD [4], among which >100 impact synaptic functions or interact with genes involved in neuronal development [5]. As a possible neurobiological substrate, clinical and animal studies point to molecular, neurodevelopmental and functional changes of deep-layer cortical projection neurons (CPNs), in particular those of layer 5 (L5) forming the corticostriatal pathway [6–9]. In this context, we have linked heterozygous *TSHZ3* gene deletion to a syndrome characterized by neurodevelopmental disorders including autistic behavior, cognitive disabilities and language disturbance, with some patients also showing renal tract abnormalities [10]. *TSHZ3* encodes the highly conserved, zinc-finger homeodomain transcription factor TSHZ3, and has been identified in networks of human neocortical genes highly expressed during late fetal development, which are involved in neurodevelopmental and neuropsychiatric disorders [9, 10]. It is now ranked as a high-confidence risk gene for ASD (https://gene.sfari.org/database/human-gene/TSHZ3#reports-tab). In human and mouse, high *TSHZ3* gene or protein expression is detectable in the cortex during pre- and postnatal development [11]. We showed that heterozygous deletion of *Tshz3* (*Tshz3*^*+/lacZ*^) and early postnatal conditional knockout (KO) using the *Camk2a-Cre* promoter (*Camk2a-cKO* mice) lead to ASD-relevant behavioral deficits paralleled by changes in cortical gene expression and corticostriatal synaptic abnormalities [10, 12]. These data suggest that *Tshz3* plays a crucial role in both pre- and postnatal brain development and functioning, and point to CPNs, and in particular to the corticostriatal pathway, as a main player in the *Tshz3*-linked ASD syndrome. In the mouse striatum, TSHZ3 is not expressed in striatal spiny projection neurons (SSPNs), which represent >90% of striatal neurons, but in a small population of cells that are likely interneurons [10]. We [13] and others [14, 15] identified these cells as being mainly striatal cholinergic interneurons (SCINs), whose implication in ASD has been suggested by some studies [16, 17]. We also showed that the *Camk2a-Cre* transgene is unexpectedly expressed in the SCIN lineage, where it efficiently elicits the deletion of *Tshz3* in *Camk2a-Cre* mice [13]. Together, these data demonstrate that, within the corticostriatal circuitry, *Tshz3* is deficient in both CPNs and SCINs, in *Tshz3*^*+/lacZ*^ heterozygous [10] as well as in *Camk2a-cKO* mice [12], which both show the full repertoire of ASD-like behavioral defects. Here, we aimed at investigating the respective contribution of CPNs and SCINs to the pathophysiology of *Tshz3*-linked ASD using targeted conditional deletion of this gene, and provided evidence for the complementary implication of these two neuronal populations in the ASD-related core features.

## Results

### Conditional deletion of *Tshz3* in CPNs

**Fig. 1:**
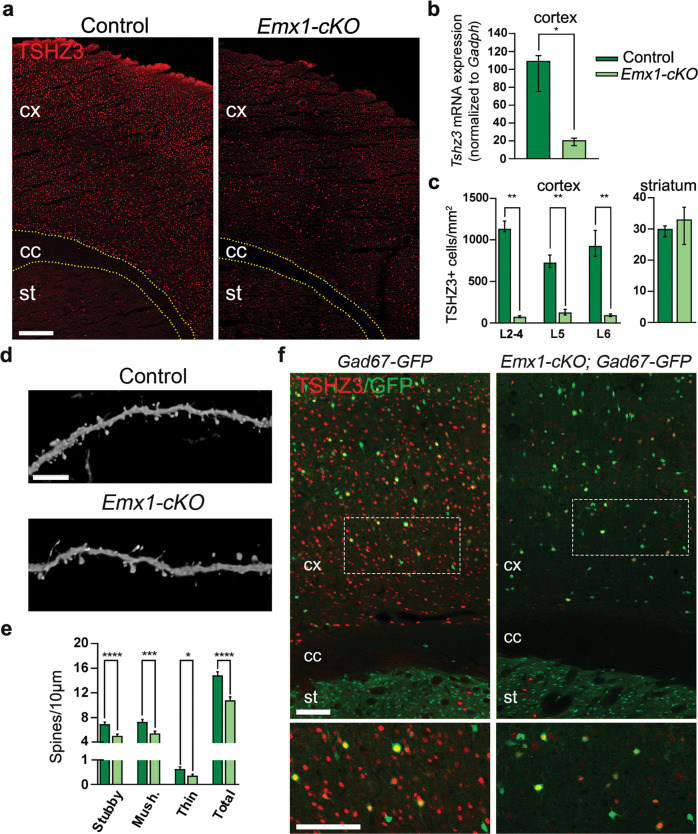
Conditional *Tshz3* deletion in CPNs. **a** Coronal brain sections from control and *Emx1-cKO* mice immunostained for TSHZ3. Scale bar 250 µm. **b** *Tshz3* mRNA relative expression in the cortex of control and *Emx1-cKO* mice measured by RT-qPCR (4 cortices per group; *P < 0.05, Mann–Whitney test). **c** TSHZ3-positive cell density in control and *Emx1-cKO* mice in cortical layers (cell counts performed using frames of 400 μm width spanning from L1 to L6 in 9 sections from 3 control mice and 18 sections from 3 *Emx1-cKO* mice; **P < 0.01, Mann–Whitney test) and in the whole striatal surface (cell counts performed in the whole dorsal striatum in 6 sections from 3 control mice and 7 sections from 3 *Emx1-cKO* mice; *P* = 0.1496, Mann–Whitney test). **d** Representative confocal images showing dendritic spines of GFP-positive L5 neurons from control (*Thy1-GFP-M*) and *Emx1-cKO* (*Thy1-GFP-M;Emx1-cKO)* mice. Scale bar 5 µm. **e** Density of different classes of dendritic spines in control (1688 spines/1135 µm) and *Emx1-cKO* (1308 spines/1220 µm) mice. **f** Coronal brain sections from *GAD67-GFP* control and *Emx1-cKO*-*GAD67-GFP* mice immunostained for TSHZ3. Lower panels are magnifications of the framed areas in the upper images. Scale bars 100 µm. *P < 0.02, ***P < 0.001 and ****P < 0.0001, Student’s *t* test. Data in (**b**) and (**c**) are expressed as medians with in*t*erquartile range; data in (**e**) are expressed as means + SEM.

High levels of *Tshz3* gene or TSHZ3 protein expression are detectable in the mouse cortex during pre- and postnatal development [10, 11]. In the adult cerebral cortex, TSHZ3 is detected in the great majority of CPNs [10]. Here, performing immunostaining for beta-galactosidase (ß-Gal) to report the expression of *Tshz3*, we show that *Tshz3* is also expressed in 26.8 ± 1.3% (*n* = 20 sections from 3 mice) of cortical GABAergic interneurons, as evidenced using *Tshz3*^*+/lacZ*^*;GAD67-GFP* mice (Fig. S1a); the percentage of dually labeled cells is significantly higher in the deep vs. upper cortical layers (36.5 ± 1.6% vs. 20.0 ± 1.0%, respectively; *P* < 0.0001, Student’s *t* test). In contrast, ß-Gal is not detectable in cortical choline acetyltransferase (CHAT) positive neurons (Fig. S1b), Olig2-positive oligodendrocytes (Fig. S1c) and GFAP-positive astrocytes (Fig. S1d, e). To address the role of *Tshz3* in CPNs, *Tshz3*^*flox/flox*^ mice were crossed with *Emx1-Cre* (*empty spiracle homeobox 1*) mice (*Emx1-cKO*). The *Emx1-Cre* mouse expresses the Cre-recombinase in the progenitors of cortical glutamatergic projection neurons (i.e., CPNs) and glial cells from embryonic day 9 (E9), but neither in those of cortical GABAergic neurons, nor of striatal interneurons, including cholinergic ones [18]. Therefore, in the corticostriatal circuitry of *Emx1-cKO* mice*, Tshz3* should be specifically lost in CPNs. Compared to control, *Emx1-cKO* mice show a drastic reduction of *Tshz3* mRNA levels and of the density of TSHZ3-positive cells in the cerebral cortex, showing the efficacy of the deletion, while the density of striatal cells expressing TSHZ3 is unchanged (Fig. 1a–c). Despite the loss of *Tshz3* expression in the vast majority of CPNs, the density of NeuN-positive cells is unchanged (Fig. S2a, b), showing no neuronal loss; in addition, neither the pattern of expression of layer-specific CPN markers, namely CUX1 for L2-4 and BCL11B for L5-6, nor the density of cells expressing these markers is affected (Fig. S2c, d), indicating no major alteration in cortical layering. However, spine density of L5 CPNs from *Thy1-GFP-M;Emx1-cKO* mice is significantly reduced compared to *Thy1-GFP-M* control mice (Fig. 1d, e). By crossing *Emx1-cKO* with *GAD67-GFP* mice, we show that cortical GABAergic neurons still express TSHZ3 (Fig. 1f), confirming the specificity of *Tshz3* deletion in CPNs. To study whether *Tshz3* loss in CPNs could indirectly affect cortical GABAergic interneurons, we compared *GAD67-GFP* control mice (Control-*GAD67-GFP*) to *Emx1-cKO-GAD67-GFP* mutant mice. No significant changes in the number of GABAergic interneurons (Control-*GAD67-GFP*: 140.7 ± 4.9, *n* = 37 sections from 5 mice; *Emx1-cKO-GAD67-GFP*: 144.6 ± 6.1, *n* = 41 sections from 7 mice; *P* = 0.624, Student’s *t* test) and in their distribution are found (Fig. S3a, b). CHAT immunostaining on striatal slices in *Emx1-cKO* mice also shows no significant modification of the density of SCINs (Fig. S3c, d). Overall, these data show that *Tshz3* is specifically lost in CPNs of *Emx1-cKO* mice, with no major consequences on the number and layer distribution of CPNs and GABAergic neurons, but with a significant reduction of L5 CPN dendritic spine density, suggesting altered synaptic communication.

### Cortical excitatory synaptic transmission and corticostriatal synaptic plasticity in *Emx1-cKO* mice

We then examined whether the loss of *Tshz3* in CPNs affects their electrophysiological properties and synaptic transmission. We recorded L5 CPNs, which are at the origin of the corticostriatal pathway, in slices from *Emx1-cKO* mice. They show no significant changes in their membrane properties and excitability compared to control (Fig. S4a–e). Action potential (AP)-dependent glutamate release onto L5 CPNs, evaluated by measuring paired-pulse ratio, is also unaffected (Fig. S4f). However, both NMDA/AMPA ratio (Fig. S4g) and NMDA-induced currents (Fig. S4h) are significantly reduced, suggesting decreased NMDA receptor-mediated signaling in *Emx1-cKO* mice. The amplitude of AMPA receptor-mediated miniature excitatory postsynaptic currents (mEPSCs) is similar in control and *Emx1-cKO* mice (Fig. S4i), further arguing for the implication of NMDA but not AMPA receptors. Conversely, mEPSC frequency is reduced (Fig. S4i), suggesting decreased AP-independent glutamate release onto L5 CPNs and/or reduced number of active excitatory synapses in *Emx1-cKO* mice, consistent with the decreased spine density on L5 CPNs (Fig. 1d, e).

**Fig. 2:**
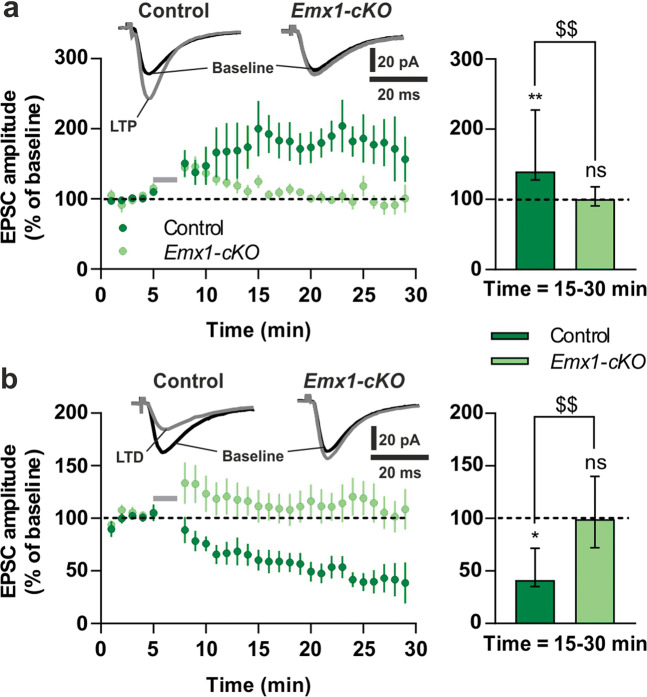
Impaired corticostriatal synaptic plasticity in *Emx1-cKO* mice. LTP (**a**) and LTD (**b**) are lost in *Emx1-cKO* mice. Left graphs: time-course (normalized EPSC amplitude expressed as means ± SEM; gray bars represent induction protocols; 2-way ANOVA from 15 to 30 min; LTP: *F*(1,211) = 44.8, *P* < 0.0001; LTD: *F*(1,216) = 153.2, *P* < 0.0001). Traces show EPSCs before (black) and after (gray) LTP and LTD induction protocols. Right graphs: EPSC amplitude at 15–30 min (medians with interquartile range; Wilcoxon matched-pairs signed rank test vs. baseline: *P < 0.05, **P < 0.01, ns non-significant; Mann–Whitney test: ^$$^*P* < 0.01). Data obtained from 17 SSPNs of control and 14 of *Emx1-cKO* mice.

SSPNs recorded in slices from *Emx1-cKO* mice show electrophysiological properties (Fig. S5a–d) and basal corticostriatal synaptic transmission (Fig. S5e–g) similar to control. However, both long-term potentiation (LTP) and long-term depression (LTD) at corticostriatal synapses are abolished in *Emx1-cKO* mice (Fig. 2). These findings suggest that the loss of *Tshz3* in CPNs does not impact their electrophysiological properties, but profoundly affects cortical synaptic transmission and corticostriatal synaptic plasticity, confirming a critical role of *Tshz3* in the functioning of the corticostriatal circuit.

### Conditional deletion of *Tshz3* in cholinergic neurons

Dual immunodetection of CHAT and ß-Gal in *Tshz3*^*+/lacZ*^ mice was performed to analyze the expression of *Tshz3* in brain cholinergic neuron populations. This was preferred to dual immunodetection of CHAT and TSHZ3 since the tissue fixation conditions for obtaining optimal detection of each protein are different, and also because TSHZ3 immunodetection provides weaker labeling and higher background than ß-Gal immunodetection. As reported previously [13], virtually all SCINs, both in the dorsal striatum and the nucleus accumbens, express *Tshz3* (Fig. S6a, h). In contrast, there are no or a little proportion (<30%) of ß-Gal-positive cells within CHAT-positive neurons in the components of the basal forebrain cholinergic system (medial septal nucleus, diagonal band nuclei, *nucleus basalis* of Meynert and *substantia innominata*) (Fig. S6a–d, h). SCINs thus represent the major population of *Tshz3*-expressing cells among the forebrain cholinergic neurons. In addition, there is almost no co-expression of ß-Gal and CHAT in the pedunculopontine (Fig. S6e, f, h) and laterodorsal tegmental nuclei (Fig. S6g, h), which are known to provide cholinergic afferents to several brain areas including the striatum [19]. Among the other brainstem nuclei, co-expression ranges from poor to extensive, as illustrated in the parabigeminal nucleus and the oculomotor nucleus, respectively (Fig. S6e, f, h).

**Fig. 3:**
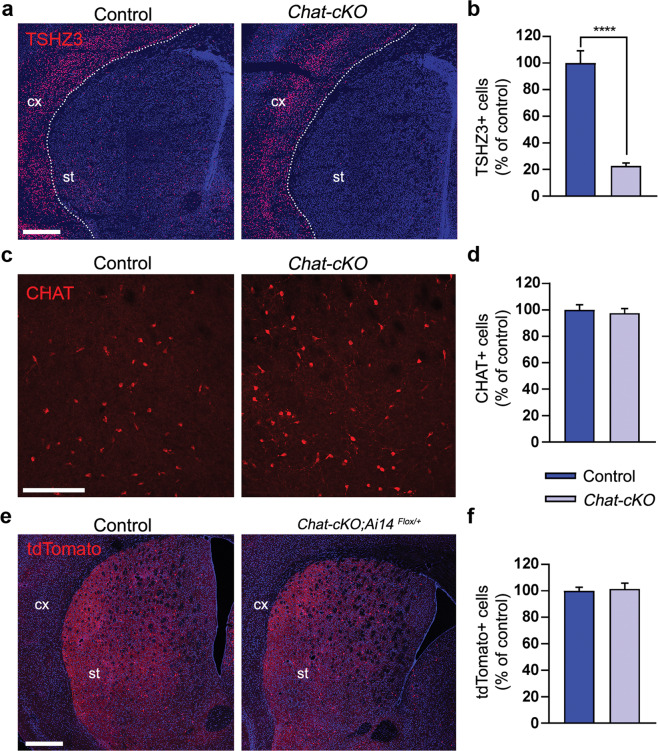
Conditional *Tshz3* deletion in cholinergic neurons. **a** Coronal brain sections from control and *Chat-cKO* mice immunostained for TSHZ3 and counterstained with DAPI. Scale bar 500 µm. **b** Number of TSHZ3-positive cells in the striatum of control and *Chat-cKO* mice (15 sections from 3 control mice; 11 sections from 3 *Chat-cKO* mice; ****P < 0.0001, Student’s *t* test). **c** Coronal brain sections from control and *Chat-cKO* mice stained for CHAT. Scale bar 200 µm. **d** Number of CHAT-positive SCINs in the striatum of control and *Chat-cKO* mice (40 sections from 9 control mice; 53 sections from 11 *Chat-cKO* mice; *P* = 0.6373, Student’s *t* test). **e** Representative images showing tdTomato fluorescence detection (red) in SCINs of *Chat-Cre;Ai14*^*Flox/+*^ control and *Chat-cKO;Ai14*^*Flox/+*^ mutant mice (coronal sections). cx, cerebral cortex; st, striatum. Nuclei are counterstained with DAPI. Scale bar 500 µm. **f** Number of tdTomato-positive cells in the striatum of *Chat-Cre;Ai14*^*Flox/+*^ control and *Chat-cKO;Ai14*^*Flox/+*^ mutant mice (14 sections from 3 control mice; 12 sections from 3 *Chat-cKO;Ai14*^*Flox/+*^ mice; *P* = 0.7773, Student’s *t* test). Da*t*a in (**b**), (**d**), and (**f**) are expressed as percent of mean control value and represented as means + SEM.

We previously reported that around 90% of the *Tshz3*-expressing cells in the striatum are SCINs [13]. Using *Tshz3*^*+/lacZ*^*;GAD67-GFP* mice, we found here that GABAergic neurons constitute 9.7 ± 1.3% (*n* = 21 sections from 3 mice) of the striatal *Tshz3* population. Therefore, in the striatum, *Tshz3* is expressed mainly in SCINs and in a very small fraction of GABAergic neurons; the latter are likely to be interneurons, as we previously reported that SSPNs do not express *Tshz3* [10]. To address the role of *Tshz3* in cholinergic neurons, *Tshz3*^*flox/flox*^ mice were crossed with *Chat-Cre* mice (*Chat-cKO* model). CHAT is expressed in the brain from early embryonic development and as soon as E18.5 in the striatum [20]. *Chat-cKO* mice show a marked decrease in the density of TSHZ3-positive cells, which confirms the loss of *Tshz3* in SCINs (Fig. 3a, b). The 20% TSHZ3-positive cells still observed in these mice may include a fraction of SCINs in which the Cre was inefficient and the above-mentioned GABAergic interneurons. The loss of *Tshz3* expression in *Chat-cKO* mice does not affect the number of striatal CHAT-positive cells either in the dorsal striatum (Fig. 3c, d) or in the nucleus accumbens (35.8 ± 1.1 vs. 33.8 ± 1.4 CHAT-positive cells/µm^2^ in control vs. *Chat-cKO*, respectively; 3 mice per genotype, 15 and 17 sections, respectively; *P* = 0.285, Student’s *t* test). This result was confirmed using *Chat-Cre;Ai14*^*Flox/+*^ control mice (*Chat-Cre*;*Rosa26-STOP-Tomato*) to visualize SCINs in the presence or absence of *Tshz3* (Fig. 3e, f).

### *Tshz3* loss and SCIN electrophysiological properties

**Fig. 4:**
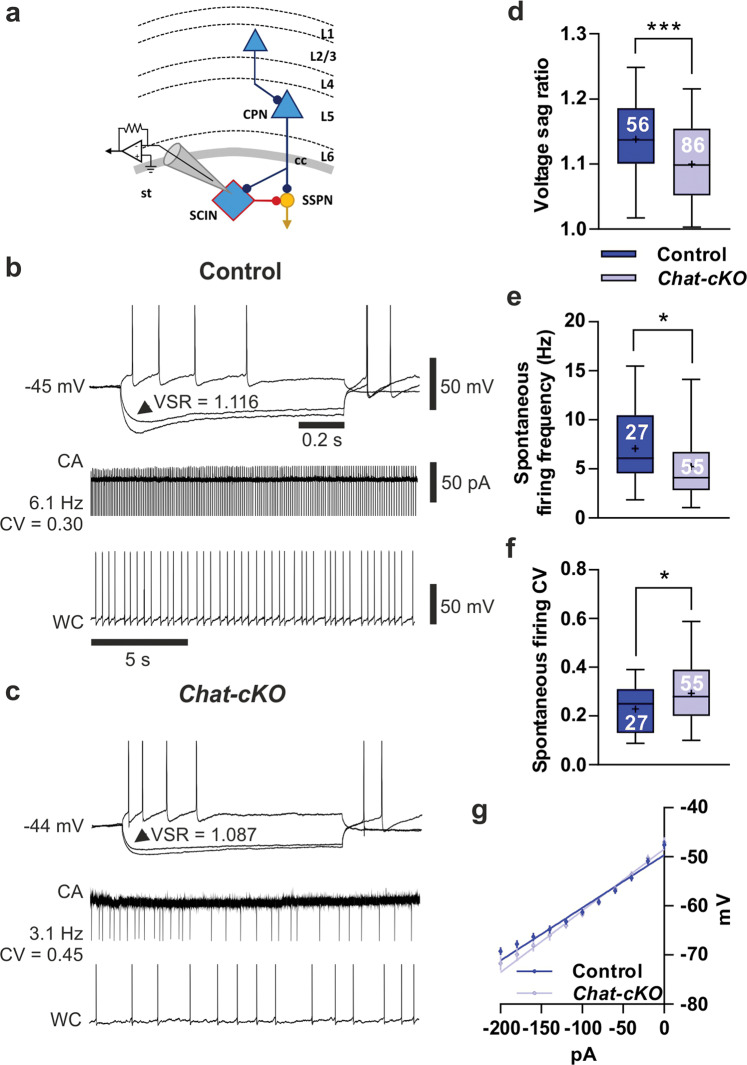
Altered electrophysiological properties of SCINs in *Chat-cKO* mice. **a** Simplified scheme of the corticostriatal circuitry with the recording patch-clamp pipette placed on a SCIN. TSHZ3-expressing neurons are blue (L1-6, cortical layers 1–6; cc, corpus callosum; st, striatum). **b** Sample traces obtained from a representative control SCIN: note the prominent voltage sag in response to −200 and −120 pA hyperpolarizing currents, and the AP firing during a + 100 pA depolarizing current (1st line), as well as the sustained and regular firing in cell-attached (CA) and whole-cell (WC) configuration (2nd and 3rd line, respectively). **c** Sample traces obtained from a representative *Chat-cKO* SCIN: compared to (**b**), note the smaller voltage sag as well as the less regular, lower frequency spontaneous firing. **b**, **c** The values of voltage sag ratio (VSR) of the response to −120 pA current injection (arrowhead), as well as the frequency and coefficient of variation (CV) of spontaneous firing of these samples, are reported; spikes have been cut; calibration bars are the same in (**b**) and (**c**). Compared to control, SCINs from *Chat-cKO* mice show a significant reduction of mean voltage sag ratio (**d)** and frequency of spontaneous discharge (**e**), while the CV of their inter-AP interval is increased (**f**) meaning that their spontaneous firing is more irregular. The number of recorded SCINs in (**d**)–(**f**) is reported in the graphs. **g** Current–voltage relationship obtained from 51 control and 62 *Chat-cKO* SCINs, and the linear best fit to calculate input resistance (see Results). *P < 0.05, ***P < 0.001, Student’s *t* test; data in (**d**–**f**) are expressed as box and whiskers (25th–75th and 5th–95th percentiles, respectively), where bar = median and cross = mean; data in (**g**) are expressed as means ± SEM.

We then determined the effect of *Tshz3* loss in cholinergic neurons on the electrophysiological properties of SCINs in the dorsal striatum. In acute brain slices, SCINs are easily recognizable among the other striatal neurons due to their larger soma [21]. Moreover, they are the only autonomously active cells, firing APs with either a regular, irregular, or bursting pattern [22, 23]. SCINs also show a characteristic depolarizing voltage sag in response to the injection of negative current pulses due to the activation of the nonspecific *I*_h_ cation current mediated by HCN channels, which largely contributes to the spontaneous AP discharge characterizing these neurons [23–25]. To test a possible effect of *Tshz3* loss on these SCIN properties, we measured the mean frequency of spontaneous AP discharge, its regularity [expressed as the coefficient of variation (CV) of the inter-AP intervals], and the amplitude of the sag [expressed as voltage sag ratio (VSR)] in SCINs from *Chat-cKO* mice and control littermates (Fig. 4a–c). We found that SCINs recorded from *Chat-cKO* mice show a significant reduction of both VSR (Fig. 4d) and spontaneous firing frequency (Fig. 4e), as well as an increased CV of inter-AP intervals that suggests a less regular discharge activity (Fig. 4f). The resting membrane potential at steady state is similar between control vs. *Chat-cKO* SCINs (46.64 ± 0.68 vs. 45.65 ± 0.64 mV, 56 vs. 86 SCINs, respectively; *P* = 0.305, Student’s *t* test), while the current–voltage relationship reveals a slight but significant increase of input resistance in *Chat-cKO* SCINs vs. control, calculated as the slope of the linear best fit (Fig. 4g; 125.7 ± 4.5 vs. 107.5 ± 4.0 MΩ, respectively; *F*(1,911) = 8.816, *P* = 0.0031). Overall, SCINs in *Chat-cKO* mice have a lower frequency and less regular AP discharge activity possibly due to a reduced *I*_h_, which could impair the physiological cholinergic tone and affect the role these neurons play in modulating striatal function.

### Conditional deletion of *Tshz3* in CPNs or in cholinergic neurons segregates the two core behavioral domains of ASD

**Fig. 5:**
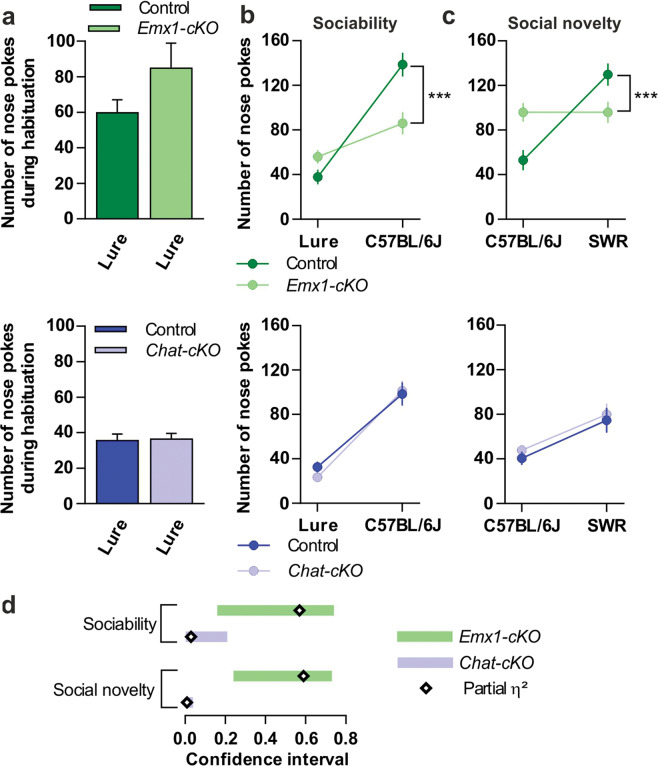
Sociability and social novelty deficits in *Emx1-cKO* but not in *Chat-cKO* mice. **a** Nose pokes during habituation, used as covariate for mixed-design ANCOVAs in (**b**) and (**c**). **b** Sociability measured as the number of nose pokes against a C57BL/6J male mouse or a lure. *Emx1-cKO* mice (*n* = 9) vs. control (*n* = 8): *F*_interaction_(1,14) = 18.59, *P* < 0.001. *Chat-cKO* mice (*n* = 12) vs. control (*n* = 9): *F*_interaction_(1,18) = 0.55, *P* = 0.47. **c** Interest in social novelty measured as the number of nose pokes against the same C57BL/6J or a SWR mouse. *Emx1-cKO* vs. control: *F*_interaction_(1,14) = 19.70, *P* < 0.001. *Chat-cKO* vs. control: *F*_interaction_(1,18) = 0.02, *P* = 0.89. **d** Sizes of the difference for *Emx1-cKO* (partial η^2^ = 0.57 and 0.59 for (**b**) and (**c**), respectively) and *Chat-cKO* mice (partial η^2^ = 0.03 and 0.001, respectively) vs. their respective control. Data in (**a**–**c**) are expressed as means ± SEM. ***P < 0.001.

**Fig. 6:**
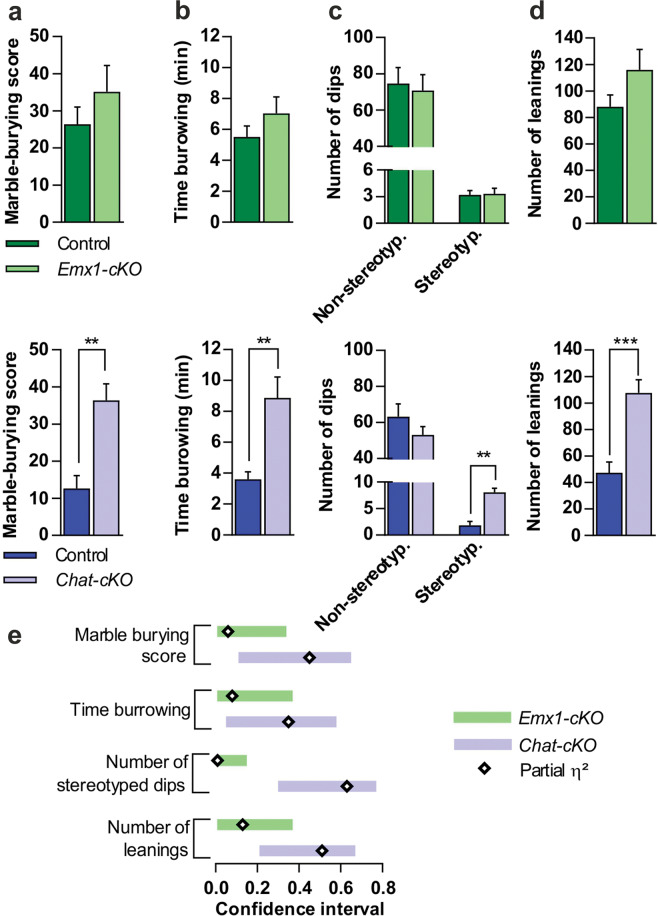
Repeated patterns of behavior in *Chat-cKO* but not in *Emx1-cKO* mice. **a** Marble-burying, *Emx1-cKO*, Student’s-*t*(15) = 1.0, *P* = 0.33; *Chat-cKO, t*(19) = 3.97, *P* = 0.001. **b** Time burrowing, *Emx1-cKO, t*(15) = 1.16, *P* = 0.13); *Chat-cKO, t*(19) = 3.225, *P* = 0.004. **c** Stereotyped dips, *Emx1-cKO, F*_*interaction*_(1,15) = 0.08, *P* = 0.87 (with non-stereotyped dips as covariate, *P* = 0.76); *Chat-cKO, F*_interaction_(1,19) = 32.69, *P* = 0.00001 (with non-stereotyped dips as covariate, *P* = 0.24). **d** Number of leanings, *Emx1-cKO, t*(15) = 1.51, *P* = 0.15; *Chat-cKO*, *t*(18) = 4.35, *P* = 0.0003. **e** Sizes of the difference in *Emx1-cKO* (η^2^ = 0.06, 0.08, 0.13 in (**a**), (**b**) and (**d**), respectively, and partial η^2^ = 0.01 in (**c**) and in *Chat-cKO* (η^2^ = 0.45, 0.35, 0.51 in (**a**), (**b**) and (**d**), respectively, and partial η^2^ = 0.63 in (**c**). Sample size of (**a**–**d**) were: 9, 9, 9, and 12 for *Emx1-cKO*; 8, 8, 9, and 11 for their controls; 12, 12, 12, and 11 for *Chat-cKO;* 9, 9, 11, and 8 for their controls. Data in (**a**–**d**) are expressed as means + SEM. **P < 0.01 ***P < 0.001.

As altered physiology of the corticostriatal circuit is postulated to play a central role in the pathophysiology of ASD, we characterized *Emx1-cKO* and *Chat-cKO* mice for ASD-relevant phenotype using a battery of behavioral tests [3] after having verified that these mice do not present visual, auditory and olfactory impairment (Fig. S7). They were tested for deficits in social behavior, the first core feature of ASD, as well for stereotyped/repetitive patterns of behavior and for restricted field of interests, which are subcategories of the second ASD core feature. During the habituation phase in the two-chamber test, both *Emx1-cKO* and *Chat-cKO* mice show no significant differences in their exploration of the lured boxes as compared to their respective controls (*P* = 0.14, η^2^ = 0.12, *P* = 0.84, η^2^ = 0.002, respectively Fig. 5a). However, *Emx1-cKO* but not *Chat-cKO* mice show impaired social relationships (Fig. 5). *Emx1-cKO* mice have less preference than their controls for a conspecific (sociability, Fig. 5b) and for an unfamiliar male (social novelty, Fig. 5c), the interaction between genotype and box content being large in each case, as shown by the effect size that exceeds the typical range of variation (Fig. 5d). Conversely, *Chat-cKO* but not *Emx1-cKO* mice present more stereotyped or repetitive patterns of behavior than their controls, as shown by the marble-burying score, time burrowing in a new cage, stereotyped dips on a hole board, and number of leanings in an open field (Fig. 6a–d), with a large effect size (Fig. 6e). Restricted field of interest is impacted neither in *Emx1-cKO* nor in *Chat-cKO* mice (Fig. S8a–c).

Finally, since impairment of motor control and learning have been reported in children with ASD [26, 27], we checked *Emx1-cKO* and *Chat-cKO* mice for motor and cognitive deficits. Hind paw coordination is impaired in *Chat-cKO* but not in *Emx1-cKO* mice (Fig. S8d, e), while spatial learning ability is unaffected in both models (Fig. S8f-i).

## Discussion

Previous studies showed that haploinsufficiency or postnatal deletion of *Tshz3* results in ASD-relevant behavioral deficits and suggested altered function of the corticostriatal circuitry as a possible substrate [10, 12]. The present findings point to SCINs as an additional player in the *Tshz3*-linked ASD syndrome. They also provide evidence that targeted conditional deletion of *Tshz3* in either CPNs (*Emx1-cKO*) or cholinergic neurons (*Chat-cKO*) segregates the two core behavioral traits used to diagnose ASD, respectively social behavior deficits and repetitive behavioral patterns, suggesting that alterations in CPNs and in SCINs contribute in a complementary manner to the repertoire of behavioral deficits linked to *Tshz3* deficiency. Restricted field of interest, which defines a sub-category of the second ASD domain, was observed neither in *Emx1-cKO* nor in *Chat-cKO* mice. This suggests that the expression of this deficit in the previously characterized models of *Tshz3* deletion may involve additional players, such as the cortical and striatal GABAergic interneurons expressing *Tshz3* whose specific role remains to be determined, and/or result from the combined dysfunction of CPNs and SCINs due to the loss of *Tshz3* in both these neuronal types. Learning ability was impacted neither by *Tshz3* postnatal deletion [12], nor in *Emx1-cKO* and *Chat-cKO* models.

Among the multiplicity of circuits involved in social behavior, the literature points out the crucial role of the cortex [28, 29]. Here we focused on the sensorimotor cortex and the dorsal striatum as a model circuit that has been characterized in several ASD mouse models. However, we cannot exclude that dysfunction in the prefrontal cortex-nucleus accumbens circuitry may also be implicated in the described ASD-related phenotype, since *Tshz3* is expressed in all cortical areas and in SCINs of both the dorsal striatum and the nucleus accumbens. Corticostriatal and striatal circuit dysfunctions are associated with ASD features, both in patients and in mouse models, with CPNs and SSPNs being highly impacted by mutations of ASD-linked genes [7, 8, 10, 12, 30, 31]. There is however increasing evidence incriminating interneuron populations of the cortex and the striatum in ASD [32]. Here, we show that, in the cortex, the ASD-related *Tshz3* gene is expressed not only in CPNs but also in about a third of GABAergic interneurons, while not in cholinergic interneurons. In contrast, in the striatum, *Tshz3*-expressing cells are for their vast majority cholinergic interneurons [13] and comprise a minority of GABAergic interneurons. To disentangle the role of CPNs from that of interneurons in the ASD symptoms linked to *Tshz3* deficiency, we generated and characterized *Emx1-cKO* mice. We confirmed the specificity of *Tshz3* deletion in CPNs within the corticostriatal circuit in this model, *Tshz3* expression in cortical and striatal interneurons being maintained. In addition, no change in the numbers and positioning of these interneurons were detected. Interestingly, we found that *Emx1-cKO* mice specifically exhibit impaired social behavior and that this deficit co-segregates with altered NMDA receptor-mediated transmission in the cortex and loss of plasticity at corticostriatal synapses. Corticostriatal synaptic plasticity has been deeply characterized, but discrepancies concerning its induction protocols and the underlying molecular and cellular mechanisms [33] make it difficult to univocally interpret our results. However, since both LTD and LTP expression require pre- and postsynaptic changes, their disruption in *Emx1-cKO* mice could be attributable to cortical circuitry defects, such as the observed decrease of NMDA receptor activity in L5 CPNs [34–36]. Our findings are also in line with studies substantiating the involvement of NMDA receptor dysfunction in social deficits associated with ASD in rodent models as well as in patients [37, 38]. Finally, consistent with the literature linking ASD with changes of dendritic spine density [39], we evidence decreased spine density in L5 CPNs of *Emx1-cKO* mice, as in our previous model [12]. Overall, these data indicate that the loss of *Tshz3* in CPNs induces morphofunctional changes in these neurons and deeply affects corticostriatal plasticity, which might result in altered processing of cortical information and account for the observed social behavior deficits.

We also investigated the contribution of cholinergic neurons in the pathophysiology of *Tshz3*-linked ASD. We show that TSHZ3 is expressed in virtually 100% of SCINs of both the dorsal striatum and the nucleus accumbens, while its expression is absent or partial in the other main brain cholinergic systems. Despite their low number, SCINs have morphofunctional features that place them as key modulators of striatal microcircuits. They play a crucial role in movement control, attentional set-shifting, habit-mediated and goal-directed behavior, and selection of appropriate behavioral responses to changes in environmental contingencies, conferring behavioral flexibility [40–44]. These interneurons are also involved in basal ganglia-related pathologies such as dystonia, Parkinson’s and Huntington’s disease, Tourette’s syndrome, obsessive compulsive disorder and drug addiction [45–50]. In contrast, despite the array of data pointing to basal ganglia and to cholinergic transmission abnormalities in ASD and in ASD models [16, 51–55], to date there is little evidence showing the specific involvement of SCINs: the partial depletion of both SCINs and fast-spiking GABAergic interneurons produces stereotypy and impaired social behavior in male mice [17], while total elimination of SCINs results in perseverative behavior that extends to social behavior, rather reminiscent of neuropsychiatric conditions as Tourette’s syndrome or obsessive-compulsive disorder [56]. The present work reveals that targeted *Tshz3* deletion in CHAT-expressing neurons leads to robust stereotyped and repetitive patterns of behavior without impacting social behavior. Given the literature associating drug-induced stereotypies with abnormalities in striatal cholinergic signaling [57–59], and the co-expression of CHAT and TSHZ3 in SCINs but not in brainstem cholinergic neurons that are known to project to the striatum [19], this behavioral deficit is likely attributable to SCINs. The lack of social behavior impairment is surprising, as altered striatal physiology is assumed to be a central node mediating repetitive motor behaviors and also a range of ASD-associated behaviors, including social deficit [30]. However, the studies examining the specific involvement of SCINs in several neurodevelopmental disorders have associated altered sociability with the depletion of this interneuron population [17, 56, 60], which is not observed in *Chat-cKO* mice. Whereas the number of SCINs in these mice is unchanged, suggesting that their generation and viability are not affected, we evidenced modifications in their firing activity and electrophysiological membrane properties. This finding is an addition to the increasing amount of data stressing the complex implication of SCINs in health and diseases [61]. How the selective loss of *Tshz3* in SCINs leads to these electrophysiological changes, what are their molecular bases and what are the consequences on striatal cholinergic signaling still need to be determined. However, SCINs are important modulators of the two populations of SSPNs forming the ”direct” and ”indirect” pathways by which the striatum regulates basal ganglia outflow, whose balanced activity is determinant for appropriate action selection [42, 62]. Thus, the changes in SCIN properties observed here could alter the way they normally respond to salient stimuli and/or reward-associated cues, thereby the way they modulate the transfer of cortical information through the striatum [40, 41, 63], as observed for example after targeted deletion of the transcription factor Er81 in SCINs [44]. This could underlie the increased stereotyped behaviors observed in *Chat-cKO* mice and, possibly, also in *Tshz3*^*+/lacZ*^ [10], as well as in *CamK2a-cKO* [12] in which we recently showed that *Tshz3* is lost also in SCINs [13]. Finally, *Chat-cKO* mice do not show basal exploration deficit, similarly to *Emx1-cKO* mice, but present impaired hind paw coordination, which is in line with motor deficiencies frequently associated with ASD [64] and with a study linking partial SCIN ablation with motor incoordination [65]. Although TSHZ3 is expressed in about 25% of cholinergic neurons of the *nucleus basalis* of Meynert and the *substantia innominata*, the similarity of spatial learning curves of control and *Chat-cKO* mice suggests minor impact of *Tshz3* deletion on the function of the basal forebrain cholinergic system, which is deeply involved in learning and memory processes [66].

In conclusion, this study shows that the conditional loss of the ASD-related gene *Tshz3* in CPNs and SCINs does not affect the numbers of these neurons but induces changes in their electrophysiological and synaptic properties, paralleled by specific ASD-like behavioral defects. It provides new experimental evidence that the two behavioral domains used to diagnose ASD are independent domains that can be triggered by dysfunction in distinct neuronal subtypes. These findings may open the road to domain-specific pharmacological and behavioral therapies.

## Materials and methods

### Mouse strains and genotyping

The *Tshz3*^*lacZ*^*, Tshz3*^*flox/flox*^, *Emx1-Cre*, *Chat-Cre*, *Rosa26-STOP-lacZ* and *Ai14* (*Rosa26-STOP-Tomato*), *GAD67-GFP,* and *Thy1-GFP* mouse lines have been described previously [10, 12, 18, 67–72]. Male heterozygous Cre mice were crossed with female *Tshz3*^*flox/flox*^ to generate the two *Tshz3* conditional knockout (cKO) mice models: *Emx1-cKO* and *Chat-cKO* [18, 70]. Littermate *Emx1-Cre*^*−/−*^ and *Chat-Cre*^*−/−*^ mice were used as respective controls. Animals carrying the *Tshz3*^*flox*^ allele and *Tshz3*^*∆*^ allele were genotyped as described previously [12]. Experimental procedures were in agreement with the recommendations of the European Communities Council Directive (2010/63/EU). They have been approved by the “Comité National de Réflexion Ethique sur l’Expérimentation Animale n°14” and the project authorization delivered by the French Ministry of Higher Education, Research and Innovation. (ID numbers 57-07112012, 2019020811238253-V2 #19022 and 2020031615241974-V5 #25232). No randomization was used and no animals or samples were excluded from the different analyses performed.

### Immunohistochemistry and histology

All stains were processed on coronal brain sections of postnatal day (P) 28–34 mice. Immunostaining for TSHZ3 alone was performed on cryostat sections of brains immediately removed after anesthesia (ketamine + xylazine, 100 + 10 mg/kg, respectively, i.p.) and frozen in dry ice until use. Before incubation with the antibodies, sections were fixed with 4% paraformaldehyde (PFA) for 15 min, then washed twice for 5 min in PBS. For TSHZ3 immunostaining and GFP detection, *GAD67-GFP* mice were anesthetized (see above) and transcardially perfused with PBS. Brains were immediately dissected out, post-fixed by immersion 2 h in 4% PFA in PBS, placed in 30% sucrose in PBS overnight and frozen in dry ice until sectioning. For the other stains, mice were anesthetized (see above) and transcardially perfused with 4% PFA in 0.1 M phosphate buffer. Brains were removed and post-fixed in 4% PFA for at least 2 h before cryostat sectioning (40 µm-thick). Brain sections were washed with PBS and blocked in PBST (0.3% Triton X-100 in PBS) with 5% BSA for 1 h at room temperature. Sections were then incubated in primary antibody diluted in blocking solution (PBST, 1% BSA) overnight at 4 °C with the following primary antibodies: mouse anti-NeuN (1:500, Millipore, Mab377), rat anti-BCL11B (1:1,000, Abcam, ab18465), goat anti-CHAT (1:100, Millipore, AB144P), rabbit anti-ß-Galactosidase (1:1,000, Cappel, 599762), goat anti-CDP/CUX1 (1:200, Santa Cruz Biotechnology, C20, SC6327) and guinea-pig anti-TSHZ3 (1:2,000; ref. [67]). Sections were then washed with PBS three times and incubated overnight at 4 °C in secondary antibodies diluted 1:1,000 in blocking solution: donkey anti-rabbit Cy3, donkey anti-guinea pig Cy3 and donkey anti-goat Cy3 (Jackson ImmunoResearch Laboratories) and goat anti-mouse Alexa Fluor 488, goat anti-rat Alexa Fluor 555 and donkey anti-goat Alexa Fluor 488 (Life Technologies). Sections were counterstained by 5 min incubation in 300 µM DAPI intermediate solution (1:1,000, Molecular Probes, Cat# B34650). Section were then washed with PBS three times, mounted on Superfrost Plus slides (Fischer Scientific) and coverslipped for imaging on a laser scanning confocal microscope (Zeiss LSM780 with Quasar detection module). Spectral detection bandwidths (nm) were set at 411–473 for DAPI, 498–568 for GFP and 568–638 for Cy3; pinhole was set to 1 Airy unit. Unbiased stereological counting of NeuN, TSHZ3, CUX1, BCL11B, CHAT, ß-Gal, and GAD67-GFP-positive neurons were done from confocal images using ImageJ software (see Figure legends for frame details). Images were assembled using Photoshop 21.2.3.

Cell counts were performed blind to the genotype in the striatum (dorsal striatum and nucleus accumbens) and in the surrounding motor and sensorimotor cortex on sections spanning from bregma 0 to +1.18 mm, AP. The whole surface was analyzed for the dorsal striatum and the nucleus accumbens. For the cortex, counts were performed in frames of 400-μm width either considering the total thickness of the cortex (NeuN, ß-Gal, GAD67-GFP), the thickness of specific layers (TSHZ3, CUX1, BCL11B) or division into 10 bins of equal size for the analysis of the distribution of GAD67-GFP-positive cells and the quantification of GAD67-GFP;ß-Gal-positive cells. For the different cholinergic nuclei, the analyses were performed on sections spanning from bregma +0.5 to +1.6 mm for the nac, +0.62 to +0.38 for ms and hdb, −0.34 to −0.8 for si and nbm, +3.8 to −4.16 for 3 N, −4.16 to −4.6 for pbg and pptg and −4.72 to −5.2 for ldtg (see Fig. S6 for abbreviations).

### Morphometric and dendritic spine analysis Of L5 CPNS

We used transgenic mouse lines (P28) expressing *Thy1-GFP* (green fluorescent protein) in L5 CPNs [72]. *Thy1-GFP-M;Emx1-cKO* were obtained by crossing *Emx1-Cre;Tshz3*^*flox/flox*^ males with *Tshz3*^*flox/flox*^ females heterozygous for *Thy1-GFP*. Analysis of spine density and morphology was performed on stacks from 100 µm-thick vibratome sections (1 µm z-step) on 4 littermate pairs using a Zeiss LSM780 (Oberkochen, Germany) laser scanning confocal microscope (×63 objective NA 1.4, 0.03 µm/pixel, voxel size 0.033 µm^2^ × 0.37 µm). Spine counts were performed blind to the genotype. They were obtained from second or third-order basal dendritic branches of randomly selected L5 CPNs. Dendrites from 5 to 7 cells were analyzed per animal, providing a cumulated dendrite length > 750 µm for each genotype. Spine identification and density measures were done using NeuronStudio [73].

### RT-qPCR

Total RNA from control and *Tshz3* mutant (P28) cerebral cortex was prepared using RNeasy Plus Universal Mini Kit gDNA eliminator (*Qiagen™*) and first-strand cDNA was synthesized using iScript Reverse Transcription Supermix kit (Bio-RAD™). No blinding was done. Real-time quantitative PCR (RT-qPCR) was performed on a CFX96 qPCR detection system (Bio-RAD™) using *SYBR*^®^ *GreenER*™ qPCR SuperMixes (*Life Technologies*™). RT-qPCR conditions: 40 cycles of 95 °C for 15 s and 60 °C for 60 s. Analyses were performed in triplicate. Transcript levels were first normalized to the housekeeping gene *Gapdh*. Primer sequences used for RT-qPCR: *Gapdh* Forward: 5′ GTCTCCTGCGACTTCAACAGCA 3′; *Gapdh* Reverse: 5′ ACCACCCTGTTGCTGTAGCCGT 3′. *Tshz3* Forward: 5′ CACTCCTTCCAGCATCTCTGAG 3′; *Tshz3* Reverse: 5′ TAGCAGGTGCTGAGGATTCCAG 3′.

### Electrophysiology

Electrophysiological data were obtained from 57 *Emx1-cKO* and 44 *Emx1-Cre*^*−/−*^ control littermates, and from 16 *Chat-cKO* and 16 *Chat-Cre*^*−/−*^ control littermates, aged P21–28. No blinding was done. Procedures were similar to those described previously [10, 12, 74]. Briefly, acute coronal slices (250 µm-thick) containing cortex and striatum were cut using a S1000 Vibratome (Leica) in ice-cold solution containing (in mM): 110 choline, 2.5 KCl, 1.25 NaH_2_PO_4_, 7 MgCl_2_, 0.5 CaCl_2_, 25 NaHCO_3_, 7 glucose, pH 7.4. Slices were kept at room temperature in oxygenated artificial cerebrospinal fluid (ACSF), whose composition was (in mM): 126 NaCl, 2.5 KCl, 1.2 MgCl_2_, 1.2 NaH_2_PO_4_, 2.4 CaCl_2_, 11 glucose and 25 NaHCO_3_, pH 7.4. Electrophysiological recordings were performed in oxygenated artificial cerebrospinal fluid (ACSF) at 34–35 °C, flowing at ~2 ml/min. L5 CPNs of the primary motor and somatosensory cortex, and SSPNs and SCINs of the dorsolateral striatum were identified by infrared video microscopy and by their electrophysiological properties [75, 76]. They were recorded by whole-cell patch-clamp using borosilicate micropipettes (5–6 MΩ) filled with an internal solution containing (in mM): 125 K-gluconate, 10 NaCl, 1 CaCl_2_, 2 MgCl_2_, 0.5 BAPTA, 19 HEPES, 0.3 Na-GTP, and 1 Mg-ATP, pH 7.3 (except for NMDA/AMPA ratio experiments, see below). Electrophysiological data were acquired by an AxoPatch 200B amplifier and pClamp 10.7 software (Molecular Devices, Wokingham, UK). Series and input resistance were continuously monitored by sending 5 mV pulses, and neurons showing ≥20% change in these parameters were discarded from the analysis.

### Characterization of CPNs, SSPNs, and synaptic transmission

A stimulating bipolar electrode was placed either in the cortex at the level of L4 to activate local fibers mainly arising from L2-3 and evoke excitatory postsynaptic currents (EPSCs) in L5 CPNs, or in the *corpus callosum* to activate corticostriatal fibers and evoke EPSCs in SSPNs [12]. We did not distinguish the two main L5 CPN subtypes, i.e. intratelencephalic and pyramidal tract neurons, because they do not differ in the electrophysiological properties analyzed here [12, 77]. Glutamatergic EPSCs were recorded in the presence of 50 µM picrotoxin at a holding potential of −60 mV (CPNs) or −80 mV (SSPNs). Spontaneous miniature EPSCs (mEPSCs) were recorded in the presence of 50 µM picrotoxin and 1 µM tetrodotoxin. Current–voltage (*I–V*) relationship was obtained in current-clamp mode by injecting hyperpolarizing and depolarizing current steps (ΔI = ± 50 pA, 800 ms), and input resistance was calculated by linear regression analysis, i.e. as the slope of the linear best fit of the *I–V* relationship of each recorded neuron. Rheobase was measured as the minimal injected current (+5 pA increments) capable of eliciting an action potential (AP). For paired-pulse ratio (PPR), EPSC amplitude was measured on 6 averaged traces at each inter-pulse interval. For analyzing mEPSCs, the detection threshold (around 3–4 pA) was set to twice the noise after trace filtering (Boxcar low-pass), and only cells exhibiting stable activity and baseline were considered. For NMDA/AMPA ratio experiments, the internal solution contained (in mM): 140 CsCl, 10 NaCl, 0.1 CaCl_2_, 10 HEPES, 1 EGTA, 2 Mg-ATP and 0.5 Na-GTP, pH 7.3. The AMPA component of the EPSC was measured at the peak at a holding potential of −60 mV, while the NMDA component was measured at +40 mV and 40 ms after the stimulation artifact, when the AMPA component is negligible, as previously described [12]. Tonic NMDA currents were elicited by bath application of 50 µM NMDA for 60 s, after a stable baseline of at least 120 s; their amplitude was measured by averaging the current values of a 5 s window around the negative peak, compared to baseline; only neurons that were capable of returning to their baseline after washout were considered. EPSC amplitude for monitoring corticostriatal long-term depression and potentiation (LTD and LTP, respectively) was measured on averaged traces (6 per minute) to obtain time-course plots and to compare this parameter before (baseline) and after induction protocols. The induction protocol for corticostriatal LTD consisted of 3 trains at 100 Hz, 3 s duration, 20 s interval, at half intensity compared to baseline [78]. LTP induction protocol was identical but, during each train, neurons were depolarized to −10 mV to allow strong activation of NMDA receptors [10, 12, 79]. For a review about corticostriatal LTD and LTP see [36].

### Characterization of SCINs

The resting membrane potential (RMP) was measured at the steady state between two consecutive APs. The current–voltage relationship was calculated from the membrane response at the end of current steps from −200 to −20 pA (20 pA steps lasting 800 ms). The voltage sag ratio (VSR) was calculated from the response to a −120 pA current step as the peak voltage drop (sag) against the voltage at the end of the current pulse [80, 81]. Such relatively small current step was chosen because, with larger steps, the sag amplitude was extremely variable between different SCINs. Spontaneous AP firing was analyzed in terms of discharge frequency (expressed in Hz) and regularity; to quantify this latter parameter, we calculated the coefficient of variation (CV) of the inter-AP intervals. Note that spontaneous AP firing was analyzed only from cell-attached recordings, which were done before switching to whole-cell; in some cases, spontaneous firing was not detectable in cell-attached configuration, thus the number of samples for AP firing analyses is smaller than the whole number of recorded SCINs.

### Behavioral analysis

#### Housing conditions

Experiments were conducted blind for the genotypes in P71-87 male *Emx1-cKO* and *Chat-cKO* mice and their respective *Emx1-Cre*^*−/−*^ and *Chat-Cre*^*−/−*^ control littermates. We used males and not female mice because the ambulatory activity of females is impacted by the estrous cycle phases in rodents [82] and may bias the results of repetitive behavior measures that are partly dependent on motor activity.

Mice used in studies on social behavior are generally reared in groups of variable size and more rarely in isolation. The choice of our rearing strategy was based on the fact that the measures of social behavior in adult mice depends on the characteristics of the previous interactions that the observed male has experienced with its peers [3, 83–85]. In the rearing in group strategy, the social behaviors directed towards the tested male can vary according to the genotypes, the androgen levels and the neurotransmitter profiles of the individuals in the groups [86]. Consequently, the social behavior measured in an individual is the resultant of the individual social ability plus a component corresponding to the interactions of the individual with the other members of the group; this effect varies with the size of the group. In addition, behavioral “contamination” resulting in an impairment of sociability in wild-type mice by cohabitant KO modeling ASD was described [85]. Such undesirable effect plus the heterogeneity of the measures in mice reared in the group should contribute to avoid this strategy for testing social behavior. On the other hand, maintaining the mice socially deprived generates a specific set of agonistic reactions that prevent the measures of social abilities. To circumvent such biases, we have developed an alternative solution for years: each tested male is housed with one female mouse belonging to a single inbred strain [86]. Here, a cKO or a control male mouse was reared and maintained with CBA/H/Gnc female mice [3]. Housing was done in transparent 35 × 20 × 18 cm cages with 1-liter poplar woodchip bedding and weekly renewed enrichment (cardboard shelter). The light (07:00–19:00) was 60 lux on the ground of the cages. The temperature was 21.5 ± 0.5 °C. Behavioral tests were performed in a dedicated room, the housing cage having been transferred one hour before the beginning of the observations.

#### Assessment of sensory function

Visual, auditory, and olfactory integrity is required to ensure the validity of the behavioral data. These sensorial capacities were tested according to previously described protocols [3, 10, 12, 87].

##### Visual capacities

The mouse was raised, taken by the tail, and a thin stick was approached to its eyes, without touching the vibrissae. Raising the head was scored 1 and grasping or trying to grasp the pen was scored 2. The test was administered five times and the sum of the scores recorded. Swimming towards a distant shelf in the Morris Water Maze provided an additional assessment of the visual abilities.

*Auditory capacities* were measured using Preyer’s response. It consists of a pinna twitching and going flat backwards against the head as reaction to sound. It is correlated with the average evoked auditory potential and can be considered as an indicator of auditory acuity [88–90]. Mice emit vocalizations (less than 20 kHz) and ultrasounds (above 20 kHz) in the presence of a conspecific male. For this reason, we evaluated the responses to stimulations in the ultrasound bandwidth (50 ± 0.008 kHz and 35 ± 0.010 kHz) using commercial dog whistles. The mice received 5 stimulations with each sound. We scored 1 for ear twitching and 2 for a pinna going flat backwards against the head.

*Olfactory ability* to detect an odor was evaluated by an increased time in sniffing a new odor using an olfactory habituation/dishabituation test. Non-social aromas and social odors (urines from C57BL/6J and SWR male mice) were presented individually to each mouse [91]. The trial was renewed the following day. The individual score was the median time spent.

#### ASD core features

Behaviors modeling the ASD domains as defined by DSM-5 were assessed. The tests were selected based on their strong robustness (reliability from 0.77 to 0.92) and on their high loading scores in factor analysis [3].

##### Deficit in social behavior

A two-chamber test derived from Moy et al. [92] was used to assess sociability and interest in social novelty. The setup and the protocol were detailed previously [3, 10, 12]. We used a 550 × 550 mm Plexiglas box split in a 150 × 550 mm empty chamber and a 400 × 550 mm chamber containing the two boxes (43 mm diameter, distant from 340 mm) in which the mice or the lure were placed. *Sociability* is operationally defined as the higher number of visits towards the box containing a conspecific versus the one containing a lure (an adult mouse-sized oblong gray pebble), and the *interest in social novelty* as the higher number of visits towards a novel conspecific than towards the familiar one. Loss of social interest and poor interest in social novelty are expected in mice models of ASD. Briefly, the test consisted of a three-period observation, each lasting 10 min: (1) habituation (the two boxes containing lures), (2) sociability (one box containing a lure and the other a C57BL/6J male) and (3) interest in social novelty (one box containing the same C57BL/6J and the other a new SWR male). The behaviors were video-recorded (Viewpoint-Behavior technologies) and the number of nose pokes towards the boxes was counted as measure of the number of visits [93].

##### Repeated patterns of behavior

We selected four measures that were highly loaded on the “repetitive patterns of behavior” factor in a factor analysis:[3] *marble-burying* and *time burrowing* in a new cage, *number of stereotyped dips* in a hole-board device, and *number of leanings* in an open field. The protocols used have been previously detailed [3, 10, 12]. The *marble-burying* and *time burrowing* tests quantify perseverating behavior [94, 95]. *Marble burying* consists in scoring the amount of marbles buried by each mouse in a 30 min session, using a 40 × 18 cm cage with 45 cm-thick litter and containing 20 marbles (9 mm diameter) on the surface of 70 mm thick dust-free sawdust. Completely buried, 2/3 buried and 1/2 buried marbles were scored 3, 2, and 1, respectively. The *time burrowing* test leans on spontaneous digging and pushing behavior that rodents display when placed into a new home cage. The length of time each mouse spent digging plus pushing was measured. The *number of stereotyped dips* was counted in a hole-board device, consisting of a 40 × 40 cm board with 16 equidistant holes (3.5 cm diameter) each equipped with photo-beams for detecting head dipping. Exploratory head dipping occurs when a rodent is placed on a surface with holes: the mouse puts its head once into one hole of the board. Head dipping is considered stereotyped when the head dips at least twice in the same hole within 2 s. The open-field behavior was measured in a circular open field (100 cm diameter and 45 cm high walls) brightly lighted (210 lux on the ground). The ground was virtually divided into three concentric zones of the equal surface. The distances walked and the times spent in the open field in the zones were automatically measured via the Viewpoint-Behavior technologies system (http://www.viewpoint.fr/news.php). The observation lasted 20 min. The number of leanings (rearing while leaning) on the walls of the structure was previously validated as a measure of repetitive behavior [3, 96]. The number of zones crossed is a measure of the narrowness of the field of interest. The total distance walked during the observation period served as covariate for the comparison between cKO mice and their respective controls [3].

#### Additional behavioral measures

Motor abnormalities and intellectual disability are not included among the ASD core features while having a noticeable but incomplete prevalence in ASD patients (≤79% and ~45%, respectively [64]). In this connection, two additional tests were conducted.

##### Hind paw coordination

A mouse was first trained to cross a smooth bar (50 × 5 × 5 cm) with large platforms on each extremity. The trained mouse was then placed on the central platform (3 × 5 cm) of a notched bar (100 cm) formed of 1.5 cm deep carvings regularly spaced (2 cm). The task consisted of ten bar crossings from the central to an extremity platform. The experimenters on each side of the setup counted the left and right hind paw slips according to [97].

##### Spatial learning

The Morris water maze provides measures of the ability of rodents to solve spatial learning problems, namely the ability to find a submerged resting platform concealed beneath opaque water. The platform is a glass cylinder (66 mm diameter, 9 mm beneath the surface of the water) positioned 23 cm from the edge of a 100 cm diameter circular tank filled with water at 26 ± 1 °C and the light at 70 lux on the surface. Each mouse performed 7 blocks of 4 trials each: one block on day 1, and two blocks daily (one in the morning and one in the afternoon) for 3 successive days. A trial was stopped after 90 s if the mouse failed to reach the platform. We considered that the mouse had reached the platform when it stayed on the platform for 5 s at least. We presented a small metal shelf to the mouse 5 cm above the platform at the end of each trial of the first block (shaping). The mouse climbed on it and was transferred to a cage with dry sawdust for 120 s. We had previously assigned 4 virtual cardinal points to the tank, each being the starting point for a trial. The starting point for each trial was chosen randomly and within a block the mouse never started more than once from the same virtual cardinal point. We measured (1) the time to reach the hidden platform and (2) the cumulative distance to the center of the platform during swimming. The second measure eliminates possible bias resulting from floating during the trial. The time to reach the platform and the distance were automatically measured by a video tracking setup (Viewpoint-Behavior technologies), each over the 7 blocks. Strains can achieve different performance levels between blocks, but without a cumulative reduction in the time to reach the platform, which is the criterion to identify the learning process. We computed the slopes of the learning curves, a negative slope indicating learning behavior [98]. The strategy was used for both the time to reach the hidden platform and the cumulative distance to the center of the platform. The probe-test procedure, conducted after removing the platform, was done 24 h after block 7 to meet the requirements for reference memory [99] and lasted 90 s. The mouse was placed in the center of the tank, and we measured the time of first crossing the virtual annulus corresponding to the location of the platform. To check whether the differences in the time to reach the platform were due to vision and/or swimming abilities rather than learning ability, we also tested groups of naïve *Emx1-cKO* and *ChAT-cKO* mice, and their respective control, to the visible platform version of the test, in which the platform is 5 mm above non-opacified water.

### Statistics

The sample size was based on previous experience and was similar to studies in the field.

#### Immunohistochemistry

Data were analyzed by Prism 7.05 (GraphPad Software, USA). Two-tailed Student’s *t* test or Mann–Whitney test was used for comparing two data sets when passing or not D’Agostino & Pearson’s normality test, respectively. Sample sizes, tests used, and *P* values are reported in Figure legends. The significance threshold was set at *P* < 0.05.

#### RT-qPCR

Statistical analysis was performed by unpaired Student’s *t* tests using the qbasePLUS software version 2 (Biogazelle). The significance threshold was set at *P* < 0.05.

#### Electrophysiology

Statistical analysis was performed by Prism 7.05 (GraphPad Software, USA). Two-tailed Student’s *t* test or Mann–Whitney test was used for comparing two data sets when passing or not D’Agostino & Pearson’s normality test, respectively. Two-way ANOVA was used to analyze the influence of 2 categorical variables. 2-samples Kolmogorov–Smirnov test was used to compare cumulative distributions. Sample sizes (n) reported in figure legends refer to the number of recorded neurons. The significance threshold was set at *P* < 0.05. The tests used, *P* values and sample sizes are indicated in the figures.

#### Behavior

Data were processed by *Statistical Package for the Social Sciences* [SPSS software, version 25 [100]]. The same statistical designs were used to compare *Emx1-cKO* and *ChAT-cKO* mice to their respective controls. Non-parametric statistics were chosen when the assumption of normality was rejected.

##### Impairment of social behavior

To analyze data from each social phase of the two-chamber test (sociability and interest for social novelty), a mixed-design analysis of covariance (ANCOVA) was used including the genotype as a fixed factor, the box content as repeated measure, with a measure of activity during habituation as covariate. A significant interaction between genotype and box content indicates that social behavior differs between the cKO and its control group.

##### Repetitive patterns of behavior and motor performance

The difference between two independent groups (cKO and its control group) was tested by an unpaired two-sample Student’s *t* test in each case where it was not necessary to include a covariate in the statistical design (i.e., stereotyped behavior: marble-burring score, time burrowing, number of leanings; motor behavior: number of hind paw slips). For measures of stereotyped dips, on which the activity level could have an impact, an analysis of covariance (ANCOVA) was performed, using the genotype as a fixed factor (cKO vs. respective control) and non-stereotyped dips as covariate.

##### Sensorial abilities

Comparison of the visual and auditory capacities of the cKO and their respective controls were conducted using a Student’s *t* test. Mixed repeated measures ANOVA, with genotype as a fixed factor and 15 odors as repeated measures, was used to compare cKO and their respective controls for olfactory capacities.

##### Spatial learning

The statistical design was the same for the time to reach the platform and the cumulative distance to the center of the platform in the Morris water maze test. Differences between the 7 blocks were tested either with Friedman’s ANOVA, a non-parametric version of one-way repeated measures ANOVA, or with two-way repeated measures mixed ANOVA design, with blocks as repeated measures variable and cKO vs. control as between-group variable. Learning may be deduced from within-bloc statistical difference and reduced time to reach the platform from one bloc to the next. The slope of the median values of the four trials in each of the seven blocks was calculated for each mouse. The median slopes for the cKO and their respective controls, as well as the time to reach the virtual platform (probe test) and the visible platform, were compared with a Student’s *t* test.

##### Effect size

Effect sizes are expressed as *η*^2^ or as partial *η*^2^ with 95% confidence interval [100, 101].

## Supplementary information

Supplementary Figure 1

Supplementary Figure 2

Supplementary Figure 3

Supplementary Figure 4

Supplementary Figure 5

Supplementary Figure 6

Supplementary Figure 7

Supplementary Figure 8

Figure legends S1-S8

## Data Availability

The data that support the findings of this study are available from the corresponding author upon reasonable request. Raw data (FastQ files) from the sequencing experiment (triplicates from wild-type and *Tshz3*-mutant striatum) and raw abundance measurements for genes (read counts) for each sample are available from Gene Expression Omnibus (GEO) under accession GSE157658, which should be quoted in any manuscript discussing the data.
