# Correction: Targeted *Tshz3* deletion in corticostriatal circuit components segregates core autistic behaviors

**DOI:** 10.1038/s41398-022-01925-x

**Published:** 2022-05-23

**Authors:** Xavier Caubit, Paolo Gubellini, Pierre L. Roubertoux, Michèle Carlier, Jordan Molitor, Dorian Chabbert, Mehdi Metwaly, Pascal Salin, Ahmed Fatmi, Yasmine Belaidouni, Lucie Brosse, Lydia Kerkerian-Le Goff, Laurent Fasano

**Affiliations:** 1 Aix-Marseille Univ, CNRS, IBDM, UMR7288 Marseille, France; 2 Aix-Marseille Univ, INSERM, MMG, UMR1251 Marseille, France; 3 Aix-Marseille Univ, CNRS, LPC, UMR7290 Marseille, France

**Keywords:** Molecular neuroscience, Autism spectrum disorders

Correction to: *Translational Psychiatry* 10.1038/s41398-022-01865-6, published online 15 March 2022

The original version of this article unfortunately contained a mistake. The figure legends of the supplemental figures were missing. The missing legends can be found below. The original article has been correct.

**Fig. S1. TSHZ3 expression in interneurons and glial cells in the cerebral cortex**. (a–e) Coronal brain sections. **a** *Tshz3* expression as β-Gal staining in *Tshz3*^*+/lacZ*^*; GAD67-GFP* mouse brain. The two images on the right are magnifications of the framed areas in A. Scale bars 100 µm. **b** Double immufluorescence staining for β-Gal and CHAT. The framed areas in (**b**) are magnified on the right. Scale bars 100 µm. **c** Double immufluorescence staining for Olig2 and ß-Gal (left) and detail of the framed area (right). Scale bars 100 µm. (**d**, **e**) Double immufluorescence staining for GFAP and ß-Gal. Scale bars 100 µm (**d**) and 50 µm (**e**). Nuclei in **c**–**e** are counterstained with DAPI. cc, corpus callosum; cx, cerebral cortex; st, striatum.

**Fig. S2. Cortical layering is preserved in** ***Emx1-cKO*** **mouse brain. a** Coronal brain sections from *Emx1-cKO* and control mice immunostained for NeuN detection. Scale bar 250 µm. **b** Number of NeuN-positive cells counted in frames of 400 μm width spanning the entire cortical thickness of control and *Emx1-cKO* mice. No genotype difference is found (11 sections from 3 mice per genotype*; P* = 0.9488, Student’s *t*-test). **c** Coronal brain sections from *Emx1-cKO* and control mice immunostained for CUX1 and BCL11B. Nuclei are counterstained with DAPI. Scale bar 100 µm; cc corpus callosum, st striatum, L layer. **d** Number of CUX1-positive cells in L2-4 and of BCL11B-positive cells in L5 and L6 in control and *Emx1-cKO* mice. No genotype difference is found (BCL11B-positive cells: 14 sections from 3 control mice and 18 sections from 3 *Emx1-cKO* mice; CUX1-positive cells: 28 sections from 4 control mice and 21 sections from 4 *Emx1-cKO* mice; counts were performed in cortical frames of 400 μm width; *P* = 0.3207 (L2/3), *P* = 0.4007 (L5) and *P* = 0.1180 (L6), Student’s *t*-test). Data are expressed as means + SEM.

**Fig. S3. Loss of** ***Tshz3*** **in** ***Emx1-cKO*** **mice does not affect the numbers of cortical GABAergic and striatal cholinergic interneurons**. Representative images **a** and quantitative analysis **b** showing the distribution of GAD67-GFP-positive cells in the cerebral cortex in coronal brain sections from *GAD67-GFP* control and *Emx1-cKO-GAD67-GFP* mice. Scale bar in A 250 µm. Data in b are expressed as percent of total GFP-positive cells per bin (37 sections from 5 control mice; 41 sections from 7 *Emx1-cKO* mice; *F*_genotype_(1100) = 0.00006, *P* = 0.994, *F*_interaction_(9100) = 0.381, *P* = 0.942, 2-way ANOVA). Images of CHAT immunostaining **c** and analysis of the density of CHAT-positive cells **d** in coronal brain sections at striatal level of control and *Emx1-cKO* mice. Scale bar 100 µm (18 sections from 3 control and 3 *Emx1-cKO* mice, respectively; *P* = 0.465, Student's *t*-test). Data in **b** are expressed as median with interquartile range; data in **d** as means + SEM.

**Fig. S4. Electrophysiological characterization of L5 CPNs and basal cortical synaptic transmission. a** Simplified scheme of the corticostriatal circuitry with the recording patch-clamp pipette placed on a L5 CPN and the stimulating electrode placed in L4. TSHZ3-expressing neurons are blue (L1-6 cortical layers 1–6, cc corpus callosum, st striatum). **b** Current-voltage relationship recorded from CPNs of *Emx1-cKO* mice and littermate controls show similar slopes and input resistance (148.9 ± 13.3 vs. 151.3 ± 11.6 MΩ, respectively; *n* = 21 and *n* = 28, respectively; *P* > 0.05, Student’s *t*-test). **c** Resting membrane potential (RMP; *n* = 28-38) and **d** rheobase (*n* = 11–21) do not significantly differ between control and *Emx1-cKO* CPNs (*P* > 0.05 for both; Student’s *t*-test and Mann-Whitney test, respectively). **e** The number of action potentials (APs) emitted by control (*n* = 10) and *Emx1-cKO* (*n* = 15) CPNs in response to increasing current injections is similar (2-way ANOVA: genotype *F*(1,138) = 3.068, *P* = 0.0821; interaction *F*(5,138) = 0.9349, *P* = 0.4605; multiple *t*-tests: *P* > 0.05). The trace shows an example of AP firing during a 100 pA, 500 ms current step. **f** Paired-pulse ratio (PPR) is not significantly different between control (*n* = 19) and *Emx1-cKO* (*n* = 14) CPNs (2-way ANOVA: genotype *F*(1,155) = 0.901, *P* = 0.344; interaction *F*(4,155) = 1.431, *P* = 0.2263). The trace shows an example of paired EPSCs (80 ms inter-pulse interval). **g** NMDA/AMPA ratio is significantly decreased in CPNs of *Emx1-cKO* mice compared to control (*n* = 15 for each genotype, **P < 0.01, Student’s *t*-test). Traces show an example of a NMDA- and an AMPA receptor-mediated EPSC recorded from the same CPN at +40 and −60 mV, respectively. **h** The tonic inward currents induced by bath application of NMDA (50 µM, 60 s) are significantly smaller in CPNs from *Emx1-cKO* mice compared to control (*n* = 15 and *n* = 14, respectively; *P < 0.05, Student’s *t*-test). The trace shows a sample response of a CPN (sEPSCs have been cut) to NMDA bath application (grey bar). **i** The distribution of mEPSC inter-event intervals is significantly different between control (*n* = 12) and *Emx1-cKO* (*n* = 11) CPNs (*P* < 0.0001, 2-samples Kolmogorov-Smirnov test), as well as their median frequency (inset) (***P < 0.001, Mann-Whitney test). Conversely, both the distribution and the median values of mEPSC amplitude are similar in control and *Emx1-cKO* CPNs (*P* > 0.05, 2-samples Kolmogorov-Smirnov test and Mann-Whitney test). Cumulative plots represent mean values (light and dark green) and SEM (grey). Traces show sample mEPSCs recorded from control and *Emx1-cKO* CPNs. Data in **b**, **c**, **e**–**h** and in **i** (cumulative plots) are expressed as means ± SEM; data in **d** and in **i** (insets) are expressed as medians with interquartile range.

**Fig. S5. Electrophysiological characterization of SSPNs and basal corticostriatal synaptic transmission. a** Simplified scheme of the corticostriatal circuitry with the recording patch-clamp pipette placed on a SSPN and the stimulating electrode placed on the cc. TSHZ3-expressing neurons are blue (L1-6, cortical layers 1-6; cc, corpus callosum; st, striatum). **b** Current-voltage relationship recorded from SSPNs of control and *Emx1-cKO* mice provide similar slopes and input resistance (97.4 ± 2.3 vs. 93.0 ± 2.1 MΩ, respectively; *n* = 7 and *n* = 15, respectively; *P* = 0.1862, Mann-Whitney test). **c** Resting membrane potential (RMP) and **d** rheobase are not significantly different between control (*n* = 7) and *Emx1-cKO* (*n* = 15) SSPNs (*P* > 0.05, Mann-Whitney test). **e** NMDA/AMPA ratio is similar between control (*n* = 11) and *Emx1-cKO* (*n* = 12) SSPNs (*P* > 0.05, Mann-Whitney test); traces in **e** show an example of an NMDA receptor- and an AMPA receptor-mediated EPSC recorded from the same SSPN at +40 and −60 mV, respectively. **f** Paired-pulse ratio (PPR) is similar between control (*n* = 18) and *Emx1-cKO* (*n* = 24) SSPNs (2-way ANOVA: genotype *F*(1162) = 0.1135, *P* = 0.7367; interaction *F*(4162) = 0.8429, *P* = 0.4999). The trace shows an example of paired EPSCs (40 ms inter-pulse interval). **g** The distribution of mEPSC inter-event intervals is significantly different between control (*n* = 8) and *Emx1-cKO* (*n* = 7) SSPNs (*P* < 0.001, 2-samples Kolmogorov-Smirnov test), but their median frequency (inset) is similar (*P* > 0.05, Mann-Whitney test). Both the distribution and the median value of mEPSC amplitude are not significantly different between control and *Emx1-cKO* SSPNs (*P* > 0.05, 2-samples Kolmogorov-Smirnov test and Mann-Whitney test). Cumulative plots represent average values (light and dark green) and SEM (grey). Traces show sample mEPSCs recorded from control and *Emx1-cKO* SSPNs. Data in **b**, **f** and **g** (cumulative plots) are expressed as means ± SEM; data in **c**–**e** and **g** insets are expressed as medians with interquartile range.

**Fig. S6. TSHZ3 expression in the main brain cholinergic systems**. Forebrain (**a**–**d**) and brainstem (**e**–**g**) coronal sections stained for ß-Gal and CHAT. (**b**, **d**, **f**) Higher-power images of framed regions in **a**, **c** and **e**, respectively. **h** Quantification of ß-Gal-positive cells within the CHAT-positive population in brain structures containing cholinergic neurons. aq, aqueduct; hdb, nucleus of the horizontal limb of the diagonal band; gp globus pallidus, ldtg laterodorsal tegmental nucleus, ms medial septal nucleus, nac nucleus accumbens, nbm nucleus basalis of Meynert, pbg parabigeminal nucleus, pptg pedunculopontine tegmental nucleus, si substantia innominata, st striatum, 3N oculomotor nucleus, 4V 4^th^ ventricle. Nuclei were counterstained with DAPI. Data are expressed as medians with interquartile range; they were obtained from 6 (3N), 7 (hdb), 9 (ms) 12 (pbg, si), 16 (ldtg), 17 (nac), 19 (st), 24 (pptg) and 40 (nbm) sections from 3 (hdb, ldtg, ms, pbg and pptg), 4 (si and 3N), 6 (nac), 7 (st) and 8 (nbm) mice, respectively.

**Fig. S7. Visual, auditory and olfactory capacities in** ***Emx1-cKO*** **and** ***Chat-cKO*** **mice compared with their respective littermate controls**. Ten mice per genotype were used in each screening. **a** Visual capacity differs neither in *Emx1-cKO* mice compared to their controls (Student’s *t* < 1, df = 18, non-significant (NS)), nor in *Chat-cKO* compared to their controls (Student’s *t* < 1, df = 18, NS). **b** Auditory capacities differ neither in *Emx1-cKO* mice compared to their controls (Student’s *t* = 1.2, df = 18, NS), nor in *Chat-cKO* mice compared to their controls (Student’s *t* < 1, df = 18, NS). **c** Time spent scenting non-social (water, violet, vanilla) and social (C57BL/6J, SWR) odors were analyzed with two mixed ANOVAs (*Emx1-cKO* and *Chat-cKO vs*. their respective control, and 15 odors as repeated measures). The genotype factor was not significant (*F* < 1, df = 1,18) in both cases. *Emx1-cKO, Chat-cKO* and their respective control spent more time sniffing social than non-social odors, as shown by comparing time sniffing vanilla 3 vs. C57BL/6J urine 1, the size of the differences being similar in each case for the KO and the control group (*Emx1-cKO* and control littermate: paired Student’s *t* = 4.5, df= 9, and *t* = 3.78, df = 9, respectively; *P* < 0.001; sizes of the differences: η^2^ = 0.57 and 0.51, respectively; *Chat-cKO* and control littermate: paired Student’s *t* = 5.7, df = 9, and *t* = 4.9, df = 9, respectively; *P* < 0.001; sizes of the differences: η^2^ = 0.49 and 0.40, respectively). Data are expressed as means + SEM. ***P < 0.001.

**Fig. S8. Restricted field of interest, hind paw coordination and spatial learning in** ***Emx1-cKO*** **vs. littermate control mice and** ***Chat-cKO*** **vs. littermate control mice***.* **a**–**c** The narrowness of the field of interest, expressed as the number of zone crossing in the open field **b** with the total distance walked **a** as covariate, is impacted neither in *Emx1-cKO* (*n* = 9) nor in *Chat-cKO* mice (*n* = 12) compared to their respective control (*n* = 8 and *n* = 8, respectively). **c** The partial η^2^ are very low and their confidence intervals includes zero. **d**, **e** Hind paw coordination. *Chat-cKO* mice (*n* = 9) exhibit a high deficit when compared to their control (*n* = 9) (Student’s *t* = 5.72, df = 16, P = 0.00003). On the opposite, *Emx1-cKO* mice (*n* = 10) do not differ from their control (*n* = 8) (Student’s *t* = 1.76, df = 16, *P* = 0.10). **e** The effect size of the difference in *Chat-cKO* (η^2^ = 0.67) exceeds the limit of impairment (0.30), whereas it is not considered in *Emx1-cKO* mice because its confidence interval encompassed zero. (**f**–**i**) Spatial learning in the Morris water maze. Time to reach the visible platform **f** is similar both in *Emx1-cKO* mice (*n* = 12) and their control (*n* = 11) and in *Chat-cKO* mice (*n* = 10) and their control (*n* = 13) (Student’s *t* = 0.90, df = 21, *P* = 0.38 and Student’s *t* = 1.28, df = 22, *P* = 0.21, respectively), showing that different learning performances cannot be attributed to motor or sensorial abilities. Non-parametric statistics were used in the hidden platform version when the assumption of normality of the distributions was rejected. We examined the learning slopes with the Friedman’s test for non-parametric ANOVA with repeated values. The four groups of mice learned across blocks 1 to 7. *Emx1-cKO* and their control learn equally (Friedman’s test for non-parametric ANOVA with repeated values: χ^2^ = 21.42, df = 6, *P* = 0.002 and χ^2^ = 19.22, df = 6, *P* = 0.004, respectively), with similar slopes (Student’s *t* = 0.01, df = 22, *P* = 0.99). *Chat-cKO* and their control also learned across blocks 1 to 7 with similar trends (χ^2^ = 24.41, df = 6, *P* = 0.0004 and χ^2^ = 30.67, df = 6, *P* = 0.00002, respectively) and similar slopes (Student’s *t* = 1.30, df = 21, *P* = 0.21). In the probe test version, the Student’s *t* in *Emx1-cKO vs*. control and *Chat-cKO vs*. controls are, respectively: Student’s *t* = 2.22, df = 22, *P* = 0.04 and Student’s *t* = 1.14, df = 21, *P* = 0.27. Dotted lines represent the 90 s cutoff. Dots indicating the visible platform values overlap. **g** The confidence intervals of the effect size for the learning slopes (η^2^ = 0.002 for *Emx1-cKO* vs. control and η^2^ = 0.07 for *Chat-cKO* vs. control) include zero, indicating that the difference of the learning slope can be disregarded. The confidence intervals of the effect size for the probe test (η^2^ = 0.17 for *Emx1-cKO* vs. control and η^2^ = 0.05 for *Chat-cKO* vs. controls) encompassed zero, indicating that the differences can be disregarded. **h** Cumulative distance from the hidden platform during the blocks. Learning was analyzed with parametric statistics (two-way mixed ANOVA with blocks as repeated-measures and cKO vs. control as between group variable). *Emx1-cKO* mice (*n*= 10) and their control (*n* = 12) learn equally (*F* = 63.18, df = 6120, *P* = 7E-35, partial η^2^ = 0.76; interaction between blocks and groups (*F* < 1), with linear trend (*F* = 209.77, df = 120, *P* = 4E-12, partial η^2^ = 0.91)) and the slopes are identical (Student’s *t =* 0.76, df = 20*, P* = 0.46, η^2^ = 0.03). *Chat-cKO* mice (*n* = 10) and their control (*n* = 11) also learn equally (*F* = 71.44, df = 6114, *P* = 2E-36, partial η^2^ = 0.79; interaction between blocks and groups (*F* < 1), with linear trend (*F* = 196.94, df = 1,19, *P* = 1E-11, partial η^2^ = 0.91)). The slopes are identical (Student’s *t* = 0.03, df = 19, *P* = 0.98, η^2^ = 0.00004). **i** The confidence intervals of the effect size for the learning slopes includes zero for both *Emx1-cKO* and *Chat-cKO vs*. their respective controls, indicating that the learning slopes do not differ in the two groups. Data are expressed as means + SEM (**a**, **b**, **d** and **h**), or as medians with interquartile range **f**. ***P < 0.001.

