# Supplementary figures and images for: Targeted Tshz3 deletion in corticostriatal circuit components segregates core autistic behaviors
Source: Transl Psychiatry. 2022 Mar 15;12:106. doi: 10.1038/s41398-022-01865-6 (PMC8924251; doi:10.1038/s41398-022-01865-6)

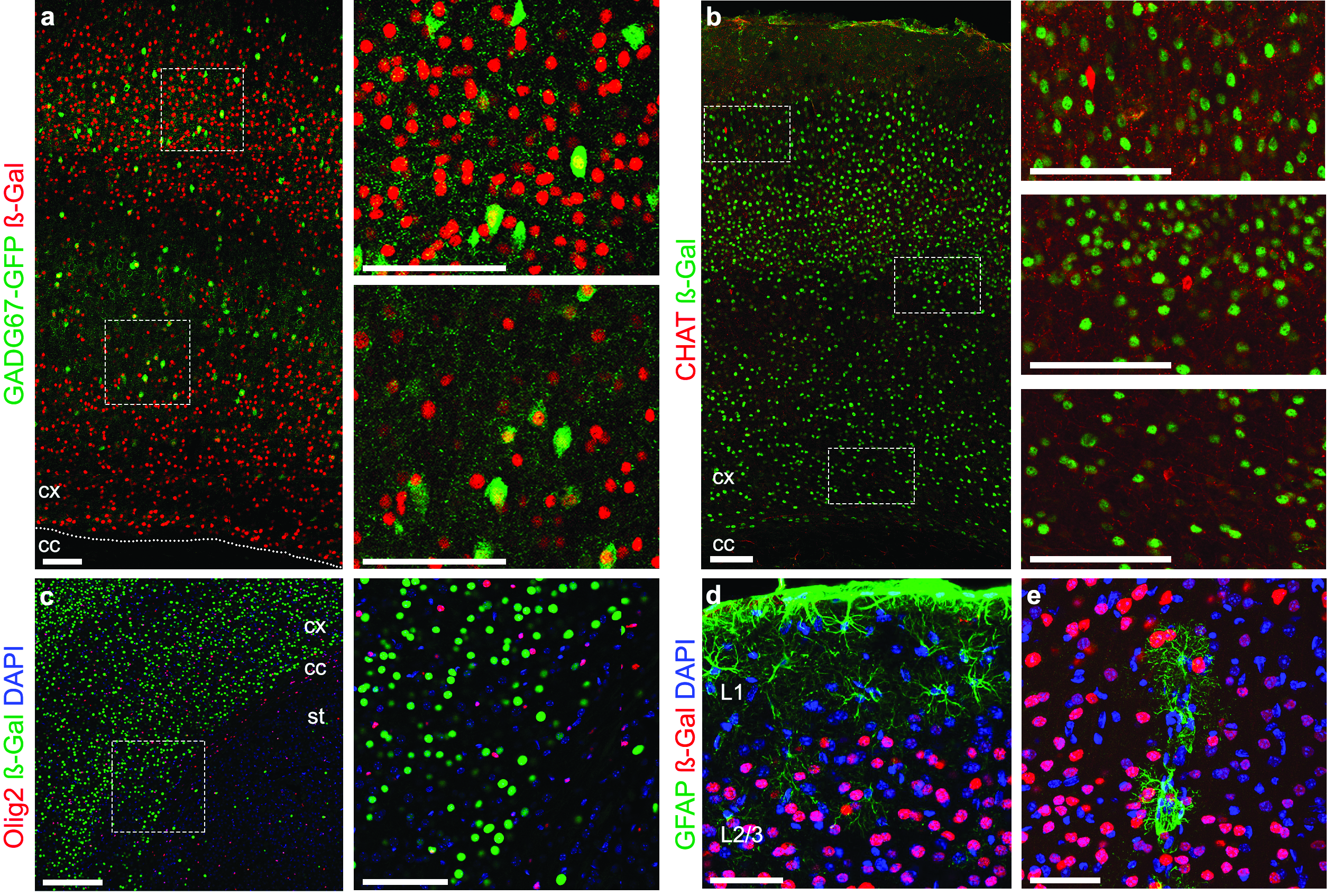

Supplement: Supplementary file 1 — Supplementary Figure 1 [file 41398_2022_1865_MOESM1_ESM.tif]

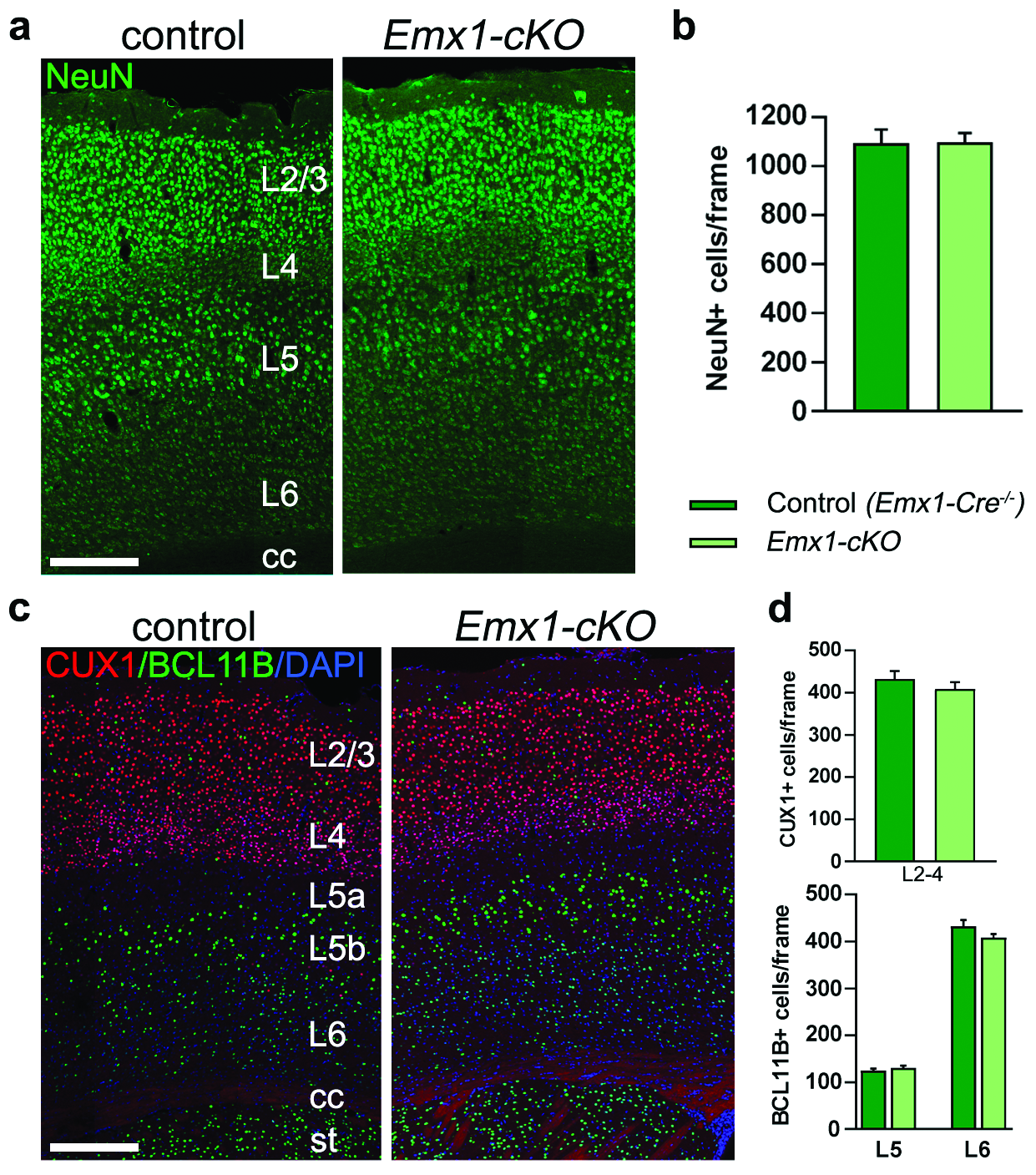

Supplement: Supplementary file 2 — Supplementary Figure 2 [file 41398_2022_1865_MOESM2_ESM.tif]

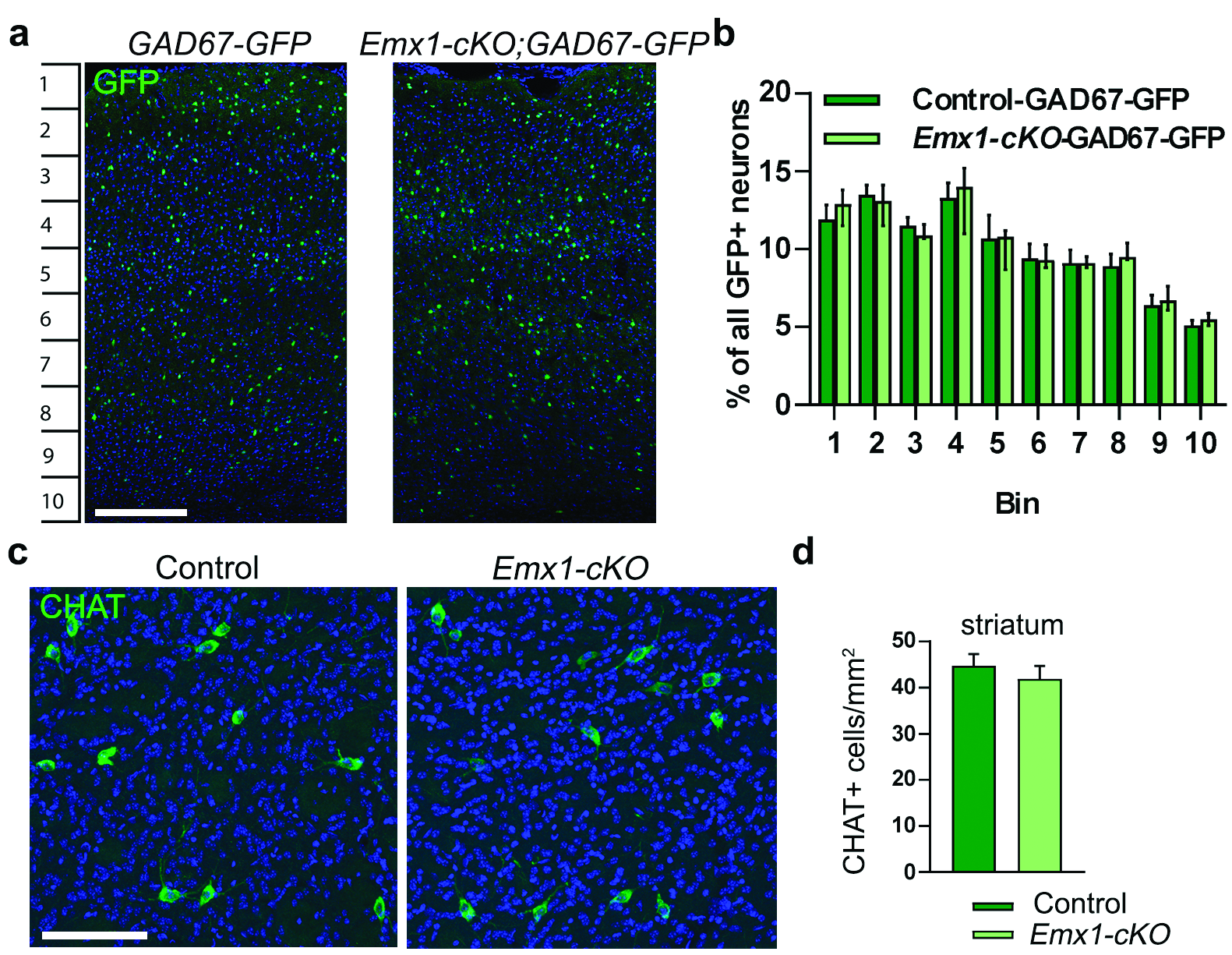

Supplement: Supplementary file 3 — Supplementary Figure 3 [file 41398_2022_1865_MOESM3_ESM.tif]

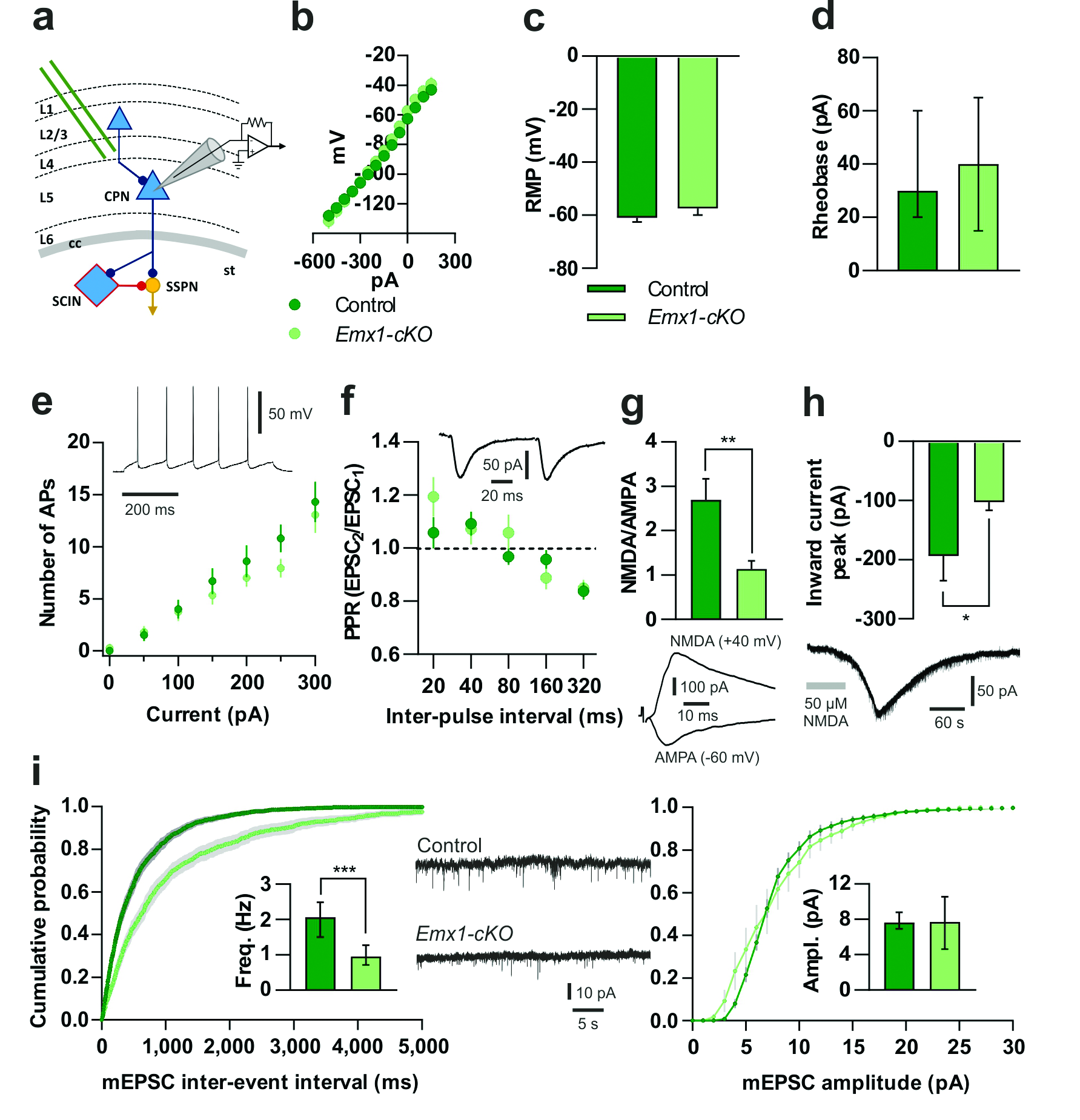

Supplement: Supplementary file 4 — Supplementary Figure 4 [file 41398_2022_1865_MOESM4_ESM.tif]

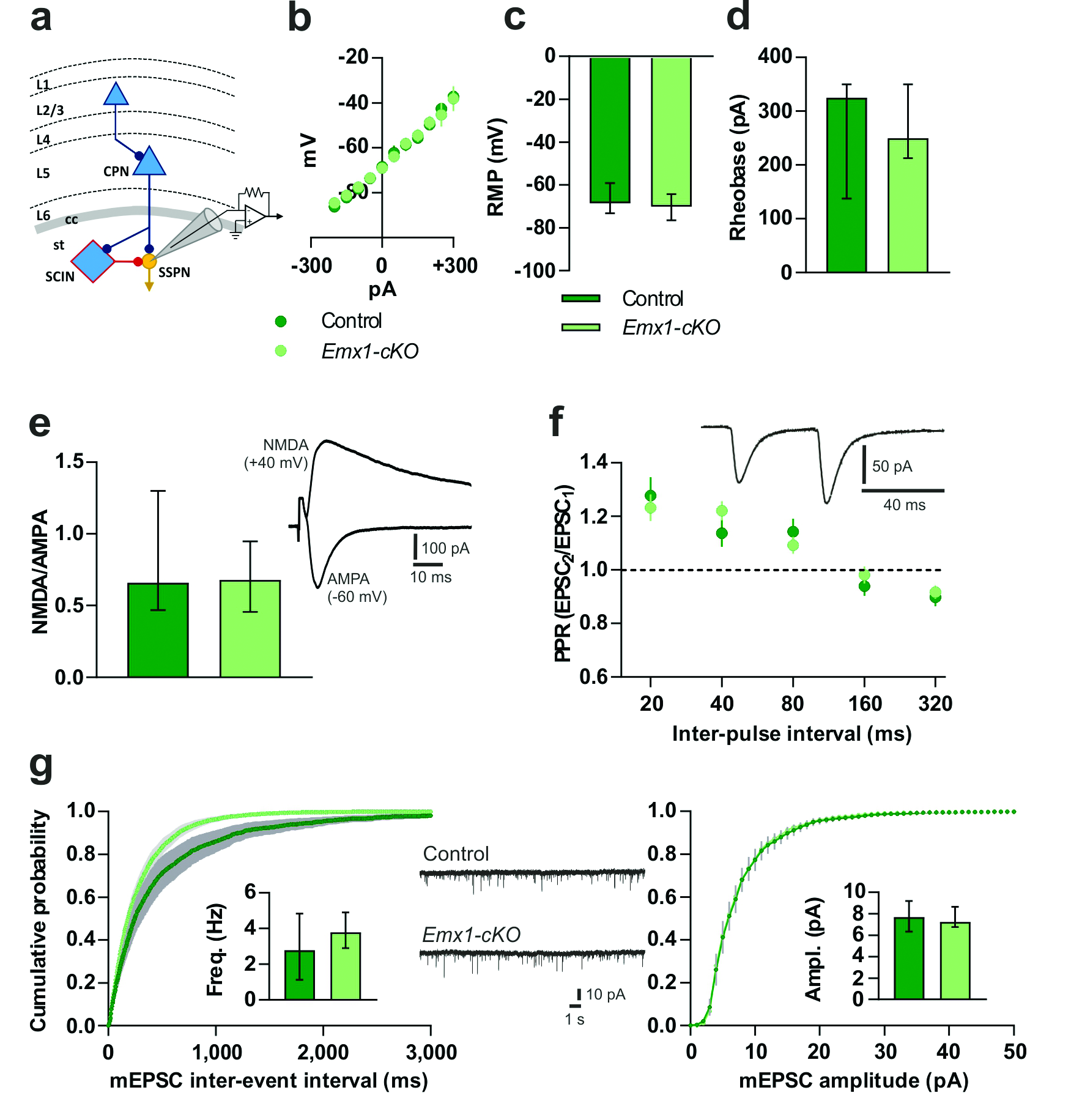

Supplement: Supplementary file 5 — Supplementary Figure 5 [file 41398_2022_1865_MOESM5_ESM.tif]

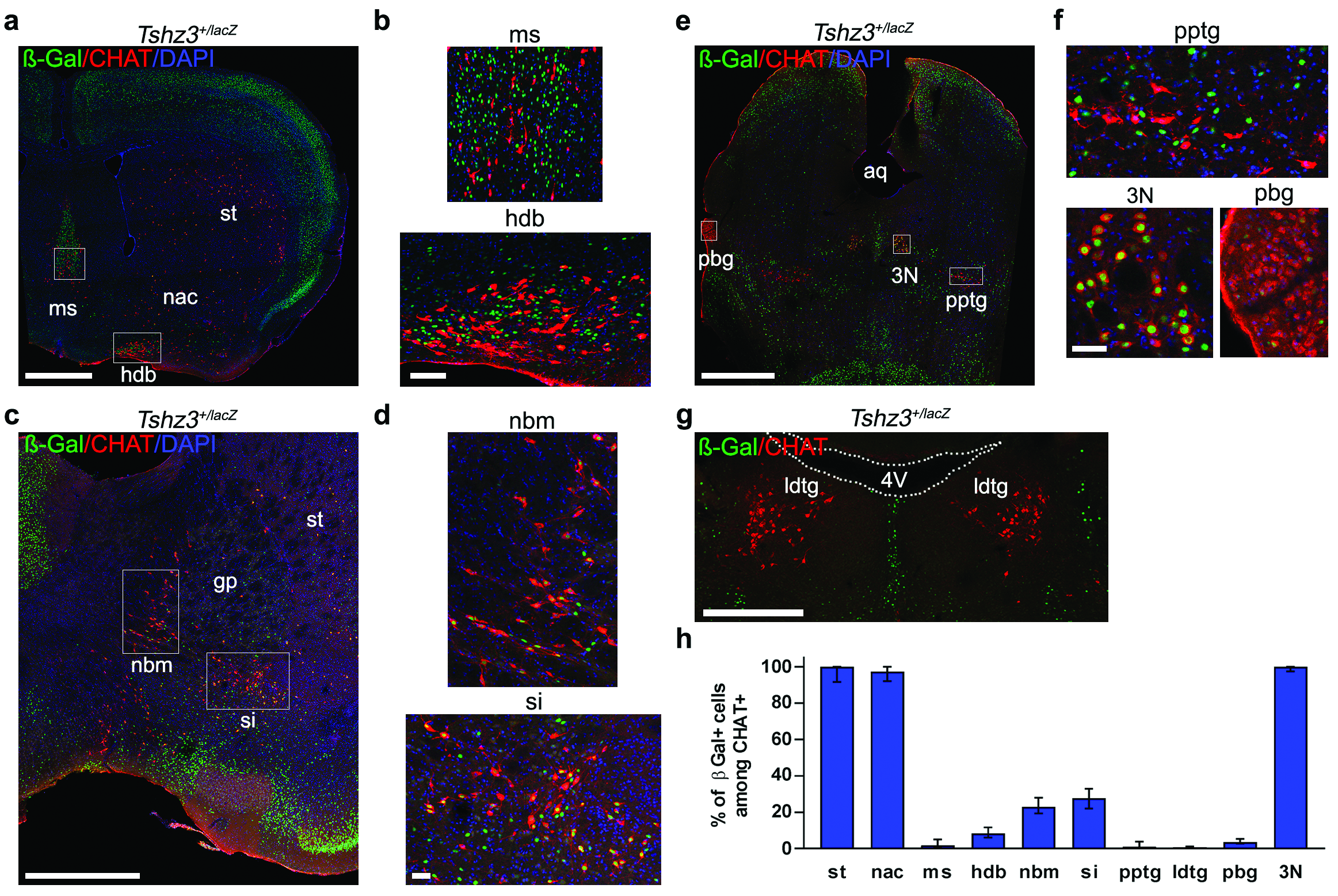

Supplement: Supplementary file 6 — Supplementary Figure 6 [file 41398_2022_1865_MOESM6_ESM.tif]

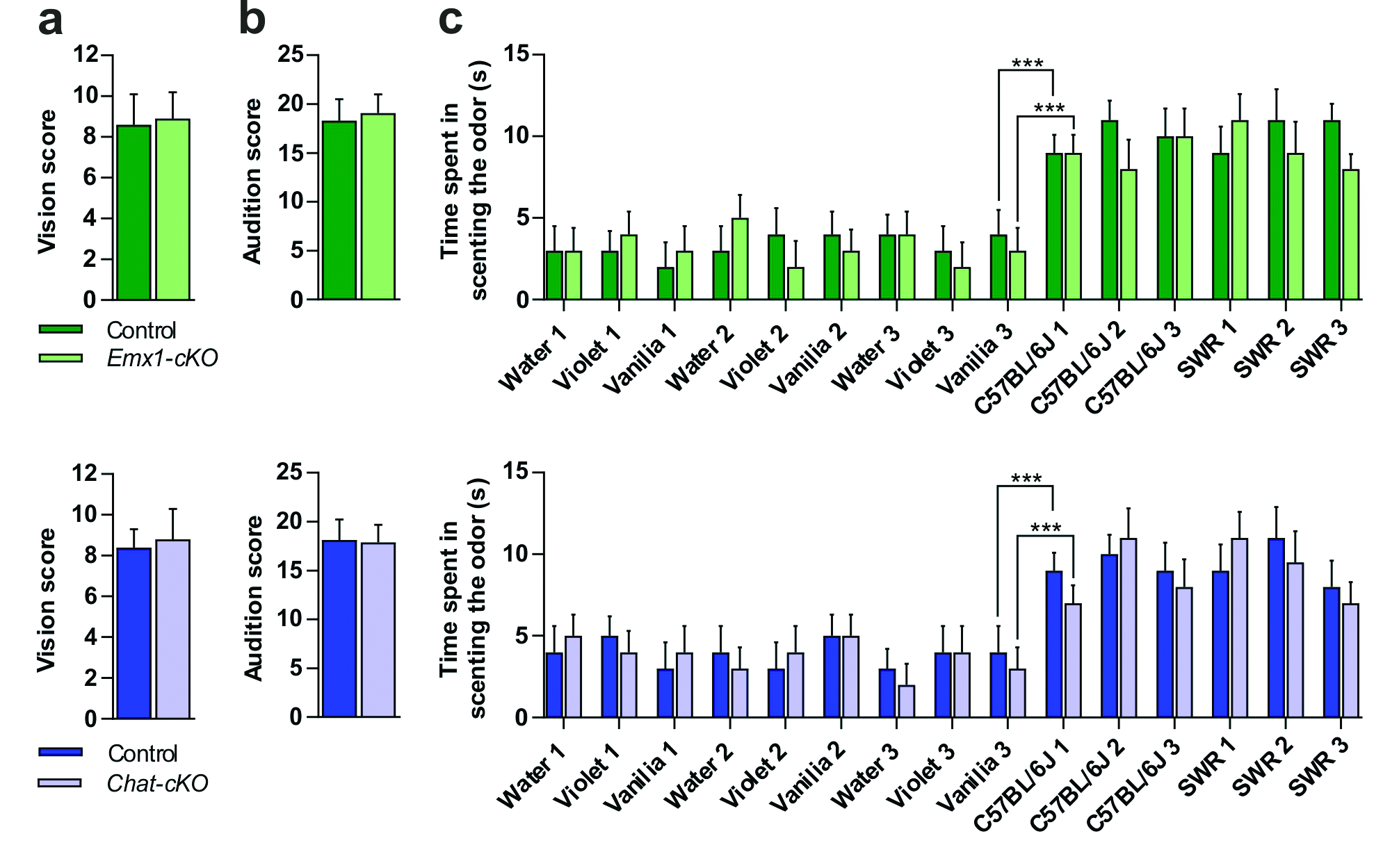

Supplement: Supplementary file 7 — Supplementary Figure 7 [file 41398_2022_1865_MOESM7_ESM.tif]

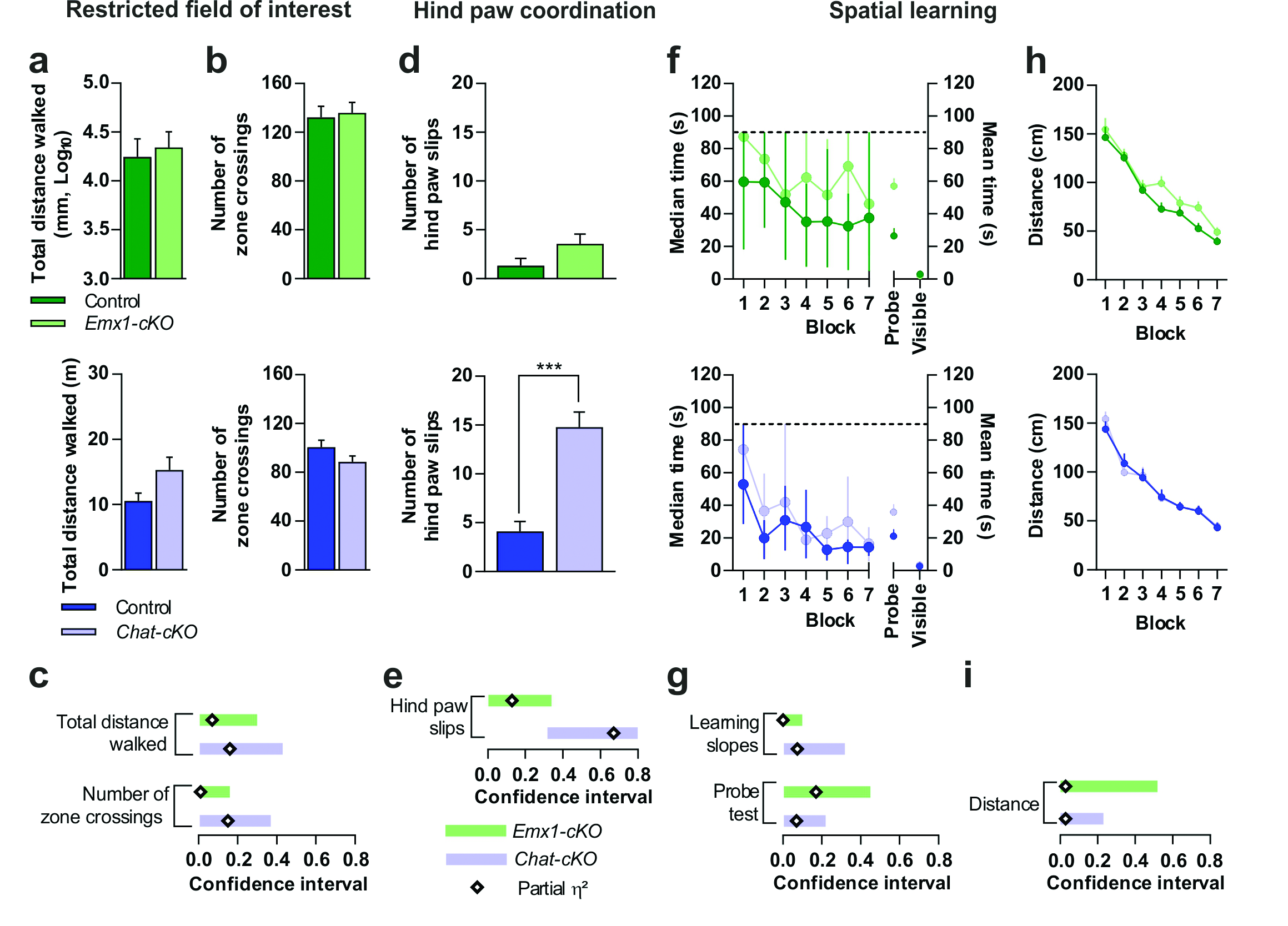

Supplement: Supplementary file 8 — Supplementary Figure 8 [file 41398_2022_1865_MOESM8_ESM.tif]
