# Supplementary material for: Targeted Tshz3 deletion in corticostriatal circuit components segregates core autistic behaviors
Source: Transl Psychiatry. 2022 Mar 15;12:106. doi: 10.1038/s41398-022-01865-6 (PMC8924251; doi:10.1038/s41398-022-01865-6)
Supplement: Supplementary file 9 — Figure legends S1-S8 [file 41398_2022_1865_MOESM9_ESM.docx]

**Fig. S1. TSHZ3 expression in interneurons and glial cells in the cerebral cortex.** (a-e) Coronal brain sections. **a** *Tshz3* expression as β-Gal staining in *Tshz3^+/lacZ^; GAD67-GFP* mouse brain. The two images on the right are magnifications of the framed areas in A. Scale bars 100 µm. **b** Double immufluorescence staining for β-Gal and CHAT. The framed areas in (**b**) are magnified on the right. Scale bars 100 µm. **c** Double immufluorescence staining for Olig2 and ß-Gal (left) and detail of the framed area (right). Scale bars 100 µm. (**d**, **e**) Double immufluorescence staining for GFAP and ß-Gal. Scale bars 100 µm (**d)** and 50 µm (**e)**. Nuclei in **c**-**e** are counterstained with DAPI. cc, corpus callosum; cx, cerebral cortex; st, striatum.

**Fig. S2.** **Cortical layering is preserved in *Emx1-cKO* mouse brain. a** Coronal brain sections from *Emx1-cKO* and control mice immunostained for NeuN detection. Scale bar 250 µm. **b** Number of NeuN-positive cells counted in frames of 400 μm width spanning the entire cortical thickness of control and *Emx1-cKO* mice. No genotype difference is found (11 sections from 3 mice per genotype*; P* = 0.9488, Student’s *t*-test). **c** Coronal brain sections from *Emx1-cKO* and control mice immunostained for CUX1 and BCL11B. Nuclei are counterstained with DAPI. Scale bar 100 µm; cc, corpus callosum; st, striatum; L, layer. **d** Number of CUX1-positive cells in L2-4 and of BCL11B-positive cells in L5 and L6 in control and *Emx1-cKO* mice. No genotype difference is found (BCL11B-positive cells: 14 sections from 3 control mice and 18 sections from 3 *Emx1-cKO* mice; CUX1-positive cells: 28 sections from 4 control mice and 21 sections from 4 *Emx1-cKO* mice; counts were performed in cortical frames of 400 μm width; *P* = 0.3207 (L2/3), *P* = 0.4007 (L5) and *P* = 0.1180 (L6), Student’s *t*-test). Data are expressed as means + SEM.

**Fig. S3. Loss of *Tshz3* in *Emx1-cKO* mice does not affect the numbers of cortical GABAergic and striatal cholinergic interneurons.** Representative images **a** and quantitative analysis **b** showing the distribution of GAD67-GFP-positive cells in the cerebral cortex in coronal brain sections from *GAD67-GFP* control and *Emx1-cKO-GAD67-GFP* mice. Scale bar in A 250 µm. Data in b are expressed as percent of total GFP-positive cells per bin (37 sections from 5 control mice; 41 sections from 7 *Emx1-cKO* mice; *F_genotype_*(1,100) = 0.00006, *P* = 0.994, *F_interaction_*(9,100) = 0.381, *P* = 0.942, 2-way ANOVA). Images of CHAT immunostaining **c** and analysis of the density of CHAT-positive cells **d** in coronal brain sections at striatal level of control and *Emx1-cKO* mice. Scale bar 100 µm (18 sections from 3 control and 3 *Emx1-cKO* mice, respectively; *P* = 0.465, Student's *t*-test). Data in **b** are expressed as median with interquartile range; data in **d** as means + SEM.

**Fig. S4.** **Electrophysiological characterization of L5 CPNs and basal cortical synaptic transmission.** **a** Simplified scheme of the corticostriatal circuitry with the recording patch-clamp pipette placed on a L5 CPN and the stimulating electrode placed in L4. TSHZ3-expressing neurons are blue (L1-6, cortical layers 1-6; cc, corpus callosum; st, striatum). **b** Current-voltage relationship recorded from CPNs of *Emx1-cKO* mice and littermate controls show similar slopes and input resistance (148.9 ± 13.3 *vs*. 151.3 ± 11.6 MΩ, respectively; n = 21 and n = 28, respectively; *P* > 0.05, Student’s *t*-test). **c** Resting membrane potential (RMP; n = 28-38) and **d** rheobase (n = 11-21) do not significantly differ between control and *Emx1-cKO* CPNs (*P* > 0.05 for both; Student’s *t*-test and Mann-Whitney test, respectively). **e** The number of action potentials (APs) emitted by control (n = 10) and *Emx1-cKO* (n = 15) CPNs in response to increasing current injections is similar (2-way ANOVA: genotype *F*(1,138) = 3.068, *P* = 0.0821; interaction *F*(5,138) = 0.9349, *P* = 0.4605; multiple *t*-tests: *P* > 0.05). The trace shows an example of AP firing during a 100 pA, 500 ms current step. **f** Paired-pulse ratio (PPR) is not significantly different between control (n = 19) and *Emx1-cKO* (n = 14) CPNs (2-way ANOVA: genotype *F*(1,155) = 0.901, *P* = 0.344; interaction *F*(4,155) = 1.431, *P* = 0.2263). The trace shows an example of paired EPSCs (80 ms inter-pulse interval). **g** NMDA/AMPA ratio is significantly decreased in CPNs of *Emx1-cKO* mice compared to control (n = 15 for each genotype, ***P* < 0.01, Student’s *t*-test). Traces show an example of a NMDA- and an AMPA receptor-mediated EPSC recorded from the same CPN at +40 and -60 mV, respectively. **h** The tonic inward currents induced by bath application of NMDA (50 µM, 60 s) are significantly smaller in CPNs from *Emx1-cKO* mice compared to control (n = 15 and n = 14, respectively; **P* < 0.05, Student’s *t*-test). The trace shows a sample response of a CPN (sEPSCs have been cut) to NMDA bath application (grey bar). **i** The distribution of mEPSC inter-event intervals is significantly different between control (n = 12) and *Emx1-cKO* (n = 11) CPNs (*P* < 0.0001, 2-samples Kolmogorov-Smirnov test), as well as their median frequency (inset) (****P* < 0.001, Mann-Whitney test). Conversely, both the distribution and the median values of mEPSC amplitude are similar in control and *Emx1-cKO* CPNs (*P* > 0.05, 2-samples Kolmogorov-Smirnov test and Mann-Whitney test). Cumulative plots represent mean values (light and dark green) and SEM (grey). Traces show sample mEPSCs recorded from control and *Emx1-cKO* CPNs. Data in **b**, **c**, **e**-**h** and in **i** (cumulative plots) are expressed as means ± SEM; data in **d** and in **i** (insets) are expressed as medians with interquartile range.

**Fig. S5. Electrophysiological characterization of SSPNs and basal corticostriatal synaptic transmission.** **a** Simplified scheme of the corticostriatal circuitry with the recording patch-clamp pipette placed on a SSPN and the stimulating electrode placed on the cc. TSHZ3-expressing neurons are blue (L1-6, cortical layers 1-6; cc, corpus callosum; st, striatum). **b** Current-voltage relationship recorded from SSPNs of control and *Emx1-cKO* mice provide similar slopes and input resistance (97.4 ± 2.3 *vs*. 93.0 ± 2.1 MΩ, respectively; n = 7 and n = 15, respectively; *P* = 0.1862, Mann-Whitney test). **c** Resting membrane potential (RMP) and **d** rheobase are not significantly different between control (n = 7) and *Emx1-cKO* (n = 15) SSPNs (*P* > 0.05, Mann-Whitney test). **e** NMDA/AMPA ratio is similar between control (n = 11) and *Emx1-cKO* (n = 12) SSPNs (*P* > 0.05, Mann-Whitney test); traces in **e** show an example of an NMDA receptor- and an AMPA receptor-mediated EPSC recorded from the same SSPN at +40 and -60 mV, respectively. **f** Paired-pulse ratio (PPR) is similar between control (n = 18) and *Emx1-cKO* (n = 24) SSPNs (2-way ANOVA: genotype *F*(1,162) = 0.1135, *P* = 0.7367; interaction *F*(4,162) = 0.8429, *P* = 0.4999). The trace shows an example of paired EPSCs (40 ms inter-pulse interval). **g** The distribution of mEPSC inter-event intervals is significantly different between control (n = 8) and *Emx1-cKO* (n = 7) SSPNs (*P* < 0.001, 2-samples Kolmogorov-Smirnov test), but their median frequency (inset) is similar (*P* > 0.05, Mann-Whitney test). Both the distribution and the median value of mEPSC amplitude are not significantly different between control and *Emx1-cKO* SSPNs (*P* > 0.05, 2-samples Kolmogorov-Smirnov test and Mann-Whitney test). Cumulative plots represent average values (light and dark green) and SEM (grey). Traces show sample mEPSCs recorded from control and *Emx1-cKO* SSPNs. Data in **b**, **f** and **g** (cumulative plots) are expressed as means ± SEM; data in **c**-**e** and **g** insets are expressed as medians with interquartile range.

**Fig. S6. TSHZ3 expression in the main brain cholinergic systems.** Forebrain (**a**-**d**) and brainstem (**e**-**g**) coronal sections stained for ß-Gal and CHAT. (**b**, **d**, **f**) Higher-power images of framed regions in **a**, **c** and **e**, respectively. **h** Quantification of ß-Gal-positive cells within the CHAT-positive population in brain structures containing cholinergic neurons. aq, aqueduct; hdb, nucleus of the horizontal limb of the diagonal band; gp, globus pallidus; ldtg, laterodorsal tegmental nucleus; ms, medial septal nucleus; nac, nucleus accumbens; nbm, nucleus basalis of Meynert; pbg, parabigeminal nucleus; pptg, pedunculopontine tegmental nucleus; si, substantia innominata; st, striatum; 3N, oculomotor nucleus; 4V, 4^th^ ventricle. Nuclei were counterstained with DAPI. Data are expressed as medians with interquartile range; they were obtained from 6 (3N), 7 (hdb), 9 (ms) 12 (pbg, si), 16 (ldtg), 17 (nac), 19 (st), 24 (pptg) and 40 (nbm) sections from 3 (hdb, ldtg, ms, pbg and pptg), 4 (si and 3N), 6 (nac), 7 (st) and 8 (nbm) mice, respectively.

**Fig. S7. Visual, auditory and olfactory capacities in *Emx1-cKO* and *Chat-cKO* mice compared with their respective littermate controls.** Ten mice per genotype were used in each screening. **a** Visual capacity differs neither in *Emx1-cKO* mice compared to their controls (Student’s *t* < 1, df = 18, non-significant (NS)), nor in *Chat-cKO* compared to their controls (Student’s *t* < 1, df = 18, NS). **b** Auditory capacities differ neither in *Emx1-cKO* mice compared to their controls (Student’s *t* = 1.2, df = 18, NS), nor in *Chat-cKO* mice compared to their controls (Student’s *t* < 1, df = 18, NS). **c** Time spent scenting non-social (water, violet, vanilla) and social (C57BL/6J, SWR) odors were analyzed with two mixed ANOVAs (*Emx1-cKO* and *Chat-cKO* *vs*. their respective control, and 15 odors as repeated measures). The genotype factor was not significant (*F* < 1, df = 1,18) in both cases. *Emx1-cKO, Chat-cKO* and their respective control spent more time sniffing social than non-social odors, as shown by comparing time sniffing vanilla 3 *vs*. C57BL/6J urine 1, the size of the differences being similar in each case for the KO and the control group (*Emx1-cKO* and control littermate: paired Student’s *t* = 4.5, df= 9, and *t* = 3.78, df = 9, respectively; *P* < 0.001; sizes of the differences: η^2^ = 0.57 and 0.51, respectively; *Chat-cKO* and control littermate: paired Student’s *t* = 5.7, df = 9, and *t* = 4.9, df = 9, respectively; *P* < 0.001; sizes of the differences: η^2^  = 0.49 and 0.40, respectively). Data are expressed as means + SEM. ****P* < 0.001.

**Fig. S8. Restricted field of interest, hind paw coordination and spatial learning in *Emx1-cKO*** ***vs*. littermate control mice and *Chat-cKO vs*. littermate control mice*.* a**-**c** The narrowness of the field of interest, expressed as the number of zone crossing in the open field **b** with the total distance walked **a** as covariate, is impacted neither in *Emx1-cKO* (n = 9) nor in *Chat-cKO* mice (n = 12) compared to their respective control (n = 8 and n = 8, respectively). **c** The partial η^2^ are very low and their confidence intervals includes zero. **d**-**e** Hind paw coordination. *Chat-cKO* mice (n = 9) exhibit a high deficit when compared to their control (n = 9) (Student’s *t* = 5.72, df = 16, P = 0.00003). On the opposite, *Emx1-cKO* mice (n = 10) do not differ from their control (n = 8) (Student’s *t* = 1.76, df = 16, *P* = 0.10). **e** The effect size of the difference in *Chat-cKO* (η^2^ = 0.67) exceeds the limit of impairment (0.30), whereas it is not considered in *Emx1-cKO* mice because its confidence interval encompassed zero. (**f**- **i**) Spatial learning in the Morris water maze. Time to reach the visible platform **f** is similar both in *Emx1-cKO* mice (n = 12) and their control (n = 11) and in *Chat-cKO* mice (n = 10) and their control (n = 13) (Student’s *t* = 0.90, df = 21, *P* = 0.38 and Student’s *t* = 1.28, df = 22, *P* = 0.21, respectively), showing that different learning performances cannot be attributed to motor or sensorial abilities. Non-parametric statistics were used in the hidden platform version when the assumption of normality of the distributions was rejected. We examined the learning slopes with the Friedman’s test for non-parametric ANOVA with repeated values. The four groups of mice learned across blocks 1 to 7. *Emx1-cKO* and their control learn equally (Friedman’s test for non-parametric ANOVA with repeated values: χ^2^ = 21.42, df = 6, *P* = 0.002 and χ^2^ = 19.22, df = 6, *P* = 0.004, respectively), with similar slopes (Student’s *t* = 0.01, df = 22, *P* = 0.99). *Chat-cKO* and their control also learned across blocks 1 to 7 with similar trends (χ^2^ = 24.41, df = 6, *P* = 0.0004 and χ^2^ = 30.67, df = 6, *P* = 0.00002, respectively) and similar slopes (Student’s *t* = 1.30, df = 21, *P* = 0.21). In the probe test version, the Student’s *t* in *Emx1-cKO vs*. control and *Chat-cKO* *vs*. controls are, respectively: Student’s *t* = 2.22, df = 22, *P* = 0.04 and Student’s *t* = 1.14, df = 21, *P* = 0.27. Dotted lines represent the 90 s cutoff. Dots indicating the visible platform values overlap. **g** The confidence intervals of the effect size for the learning slopes (η^2^ = 0.002 for *Emx1-cKO vs*. control and η^2^ = 0.07 for *Chat-cKO* *vs*. control) include zero, indicating that the difference of the learning slope can be disregarded. The confidence intervals of the effect size for the probe test (η^2^ = 0.17 for *Emx1-cKO vs*. control and η^2^ = 0.05 for *Chat-cKO* *v*s. controls) encompassed zero, indicating that the differences can be disregarded. **h** Cumulative distance from the hidden platform during the blocks. Learning was analyzed with parametric statistics (two-way mixed ANOVA with blocks as repeated-measures and cKO *vs*. control as between group variable). *Emx1-cKO* mice (n= 10) and their control (n= 12) learn equally (*F* = 63.18, df = 6,120, *P* = 7E-35, partial η^2^ = 0.76; interaction between blocks and groups (*F* < 1), with linear trend (*F* = 209.77, df = 1,20, *P* = 4E-12, partial η^2^ = 0.91)) and the slopes are identical (Student’s *t =* 0.76*,* df = 20*, P* = 0.46, η^2^ = 0.03). *Chat-cKO* mice (n = 10) and their control (n= 11) also learn equally (*F* = 71.44, df = 6,114, *P* = 2E-36, partial η^2^ = 0.79; interaction between blocks and groups (*F* < 1), with linear trend (*F* = 196.94, df = 1,19, *P* = 1E-11, partial η^2^ = 0.91)). The slopes are identical (Student’s *t* = 0.03, df = 19, P = 0.98, η^2^ = 0.00004). **i** The confidence intervals of the effect size for the learning slopes includes zero for both *Emx1-cKO* and *Chat-cKO* *vs*. their respective controls, indicating that the learning slopes do not differ in the two groups. Data are expressed as means + SEM (**a**, **b**, **d** and **h**), or as medians with interquartile range **f**. ****P* < 0.001.
